# Supplementary material for: Cost-effectiveness of internet-delivered cognitive behavioural therapy in patients with cardiovascular disease and depressive symptoms: secondary analysis of an RCT
Source: BMJ Open. 2022 Apr 10;12(4):e059939. doi: 10.1136/bmjopen-2021-059939 (PMC9002253; doi:10.1136/bmjopen-2021-059939)
Supplement: Author's manuscript [file bmjopen-2021-059939.draft_revisions.pdf]

BMJ Open is committed to open peer review. As part of this commitment we make the peer review history of every article we publish publicly available.

When an article is published we post the peer reviewers' comments and the authors' responses online. We also post the versions of the paper that were used during peer review. These are the versions that the peer review comments apply to.

The versions of the paper that follow are the versions that were submitted during the peer review process. They are not the versions of record or the final published versions. They should not be cited or distributed as the published version of this manuscript.

BMJ Open is an open access journal and the full, final, typeset and author-corrected version of record of the manuscript is available on our site with no access controls, subscription charges or pay-per-view fees (http://bmjopen.bmj.com).

If you have any questions on BMJ Open's open peer review process please email info.bmjopen@bmj.com

BMJ Open

Cost-effectiveness of Internet-delivered cognitive behavioural therapy in patients with cardiovascular disease and depressive symptoms. Secondary analysis of a RCT.

Journal:	*BMJ Open*
Manuscript ID	bmjopen-2021-059939
Article Type:	Original research
Date Submitted by the Author:	07-Dec-2021

Complete List of Authors:	Mourad, Ghassan; Linköping University, Department of health, medicine, and caring sciences<br>Lundgren, Johan ; Linköping University, Department of health, medicine, and caring sciences<br>Andersson, Gerhard; Linköping University, Department of Behavioural Sciences and Learning & Department of Biomedical and Clinical Sciences; Karolinska Institute, Department of Clinical Neuroscience<br>Husberg, Magnus; Linköping University, Department of health, medicine, and caring sciences<br>Johansson, Peter; Linköping University, Department of health, medicine, and caring sciences; Linköping University, Department of internal medicine

Keywords:	Telemedicine < BIOTECHNOLOGY & BIOINFORMATICS, HEALTH ECONOMICS, Heart failure < CARDIOLOGY, Myocardial infarction < CARDIOLOGY

SCHOLARONE™
Manuscripts

I, the Submitting Author has the right to grant and does grant on behalf of all authors of the Work (as defined in the below author licence), an exclusive licence and/or a non-exclusive licence for contributions from authors who are: i) UK Crown employees; ii) where BMJ has agreed a CC-BY licence shall apply, and/or iii) in accordance with the terms applicable for US Federal Government officers or employees acting as part of their official duties; on a worldwide, perpetual, irrevocable, royalty-free basis to BMJ Publishing Group Ltd ("BMJ") its licensees and where the relevant Journal is co-owned by BMJ to the co-owners of the Journal, to publish the Work in this journal and any other BMJ products and to exploit all rights, as set out in our licence.

The Submitting Author accepts and understands that any supply made under these terms is made by BMJ to the Submitting Author unless you are acting as an employee on behalf of your employer or a postgraduate student of an affiliated institution which is paying any applicable article publishing charge ("APC") for Open Access articles. Where the Submitting Author wishes to make the Work available on an Open Access basis (and intends to pay the relevant APC), the terms of reuse of such Open Access shall be governed by a Creative Commons licence – details of these licences and which Creative Commons licence will apply to this Work are set out in our licence referred to above.

Other than as permitted in any relevant BMJ Author's Self Archiving Policies, I confirm this Work has not been accepted for publication elsewhere, is not being considered for publication elsewhere and does not duplicate material already published. I confirm all authors consent to publication of this Work and authorise the granting of this licence.

For peer review only

Cost-effectiveness of Internet-delivered cognitive behavioural therapy in patients with cardiovascular disease and depressive symptoms. Secondary analysis of a RCT.

Ghassan Mourad PhD, RN*, Department of Health, Medicine and Caring Sciences, Linköping University, Linköping, Sweden. ghassan.mourad@liu.se

Johan Lundgren PhD, RN. Department of health, medicine, and caring sciences. Linköping University, Linköping, Sweden. johan.lundgren@liu.se

Gerhard Andersson, Professor, PhD. Department of Behavioural Sciences and Learning, Linköping University, Linköping, Sweden. Department of Biomedical and Clinical Sciences. Linköping University, Linköping, Sweden. Department of Clinical Neuroscience, Karolinska institutet, Stockholm, Sweden. gerhard.andersson@liu.se

Magnus Husberg, Department of health, medicine, and caring sciences. Linköping University, Linköping, Sweden. magnus.husberg@liu.se

Peter Johansson Professor, PhD, RN, Department of Health, Medicine and Caring Sciences and Department of internal medicine, Linköping University, Linköping, Sweden. peter.b.johansson@liu.se

***Corresponding author: Ghassan Mourad**

Address: Linköpings universitet, Kungsgatan 40, S-601 74 Norrköping

ORCID iD 0000-0001-9140-8922

Telephone: +46 11 363514

Fax: +46 11 125448

E-mail: ghassan.mourad@liu.se

Word count: 2997 (title page, abstract, references, figure legends, figures and tables excluded)

Number of tables and figures: 6

For peer review only

Abstract

Introduction: Cost-effectiveness evaluations of psychological interventions, such as Internet-delivered cognitive behavioural therapy (iCBT) programs, in patients with cardiovascular disease are rare. We recently reported moderate to large effect sizes on depressive symptoms in CVD outpatients following a 9-week iCBT program compared to an online discussion forum (ODF), in favour of iCBT [1]. In this paper we evaluate the cost-effectiveness of this intervention.

Methods: Cost-effectiveness analysis of a RCT (ClinicalTrials.gov, NCT02778074). The EQ-5D-3L was used to calculate quality-adjusted life years (QALYs). Data on healthcare costs were retrieved from healthcare registries.

Results: At 12-month follow-up, the QALY was significantly higher in iCBT compared to the ODF group (0.713 vs.0.598, *p*=.007). The mean difference of 0.115 corresponds with 42 extra days in best imaginable health status in favour of the iCBT group over the course of one year. Incremental cost-effectiveness ratio (ICER) for iCBT versus ODF was €18,865 per QALY saved. The cost-effectiveness plane indicated that iCBT is a cheaper and more effective intervention in 24.5% of the cases, and in 75% a costlier and more effective intervention than ODF. Only in about 0.5% of the cases, there was an indication of a costlier, but less effective intervention compared to ODF.

Conclusions: The ICER of €18,865 was lower than the cost-effectiveness threshold range of €23,400-€35,100 as proposed by the NICE guidelines, suggesting that the iCBT treatment of depressive symptoms in patients with CVD is cost-effective.

For peer review only

For peer review only - http://bmjopen.bmj.com/site/about/guidelines.xhtml

Strengths and limitations

This is the first cost-effectiveness study of iCBT in patients with CVD and depression

The study was based on actual costs retrieved from data registries

The study was primarily designed to evaluate the effect of iCBT and not cost-effectiveness

What is already known about this subject?

iCBT has moderate to large effect on depressive symptoms in outpatient with CVD compared to online discussion forum

What does this study add?

iCBT treatment of depressive symptoms in outpatients with CVD is cost-effective in relation to online discussion forum

How might this impact on clinical practice?

iCBT can be used as a risk factor modification tool in cardiac rehabilitation of patients with CVD and depressive symptoms

Key words

Cardiovascular disease; Cost-effectiveness. ICER, Internet-delivered cognitive behavioural therapy, Online discussion forum.

For peer review only - http://bmjopen.bmj.com/site/about/guidelines.xhtml

Introduction

Depression is found in 20%–40% of patients with cardiovascular disease (CVD) [2], and has shown to shorten life expectancy [2], induce higher risk of non-fatal cardiac events and re-hospitalization, compared to CVD patients without depression [3]. Depression in CVD is also a societal problem in the way that it increases the healthcare costs. In a study including patients with chronic stable angina, depression was a significant predictor of increased healthcare costs one year after index angiogram. The adjusted cumulative healthcare cost was 1.33, indicating a mean of 33% increase in one-year healthcare costs for those with depression [4]. In another study, Palacios et al [5] measured depression over a three-year period in outpatients with coronary heart disease and found that patients with worsening or chronic depressive symptoms had more than twice the costs than patients without depressive symptoms. However, they also found that those with depressive symptoms who were detected and treated had lower costs than those who remained untreated. The authors emphasized that interventions targeting depression and healthcare costs in outpatients are warranted [5]. In addition to this, the cost-effectiveness of psychological interventions in cardiac rehabilitation (CR) programs has been reported to be the least studied CR component [6]. In conclusion, this highlights a need for studies that perform health-economic/cost-effectiveness evaluations of psychological interventions in CVD patients.

In recent years Internet-based cognitive behavioural therapy (iCBT) has received increased attention as a treatment option for depression in CVD patients [7, 8]. One reason for this is a low access to face-to face CBT. Another reason is the Covid-19 pandemic which has increased the need to deliver care by digital solutions, in particular for persons who are susceptible of a worser outcome in Covid-19, such as those with CVD [7]. In general, for middle-aged populations iCBT has been found to decrease depression [9], but only a minority of all iCBT studies have performed cost-effectiveness analyses. Moreover, in the existing

For peer review only - http://bmjopen.bmj.com/site/about/guidelines.xhtml

studies the evidence of the cost-effectiveness of iCBT on depression is mixed [1, 10, 11]. In CVD, only a few studies have evaluated the effect of iCBT on depression [5] and to our knowledge no study has evaluated the cost-effectiveness of iCBT in CVD patients. In a recent RCT we found that a 9-week iCBT-program had moderate to large effect on depressive symptoms in CVD outpatient compared to an online discussion forum (ODF) [1]. The aim of this study was to report the cost-effectiveness of iCBT compared to ODF.

Methods

Design, setting and participants

This was a cost-effectiveness study of a randomized controlled trial (RCT) aiming to reduce depressive symptoms in patients with CVD, and that has been described in detail elsewhere [1]. In brief, patients who had been in contact with the medical or cardiac clinics at four hospitals in Southeastern Sweden were invited to participate by post. Those who consented to participate were screened for depressive symptoms (i.e., score ≥5 on the Patient Health Questionnaire-9 (PHQ-9)). In total 144 patients fulfilled the criteria, including age ≥18 years, and no hospitalizations during the past four weeks prior to inclusion, and were allocated to nine weeks of iCBT (n=72) or ODF (n=72). Thirty-eight percent (n=27) of those in the ODF group received iCBT after study completion. The iCBT program was guided by study nurses and comprised goal setting, psychoeducation, problem-solving, behavioural activation including homework assignments with weekly written feedback. In the iCBT group, 60% of the participants completed all seven modules, and 82% completed at least four. In the ODF, nine discussion topics were included and moderated by the study nurses. About 27% of those participants were active at least nine times during the intervention.

For peer review only - http://bmjopen.bmj.com/site/about/guidelines.xhtml

Health related quality of life and quality-adjusted life years

HRQoL was measured using the EQ-5D-3L (three levels), which consists of a descriptive system and the EQ visual analogue scale (EQ-VAS). The EQ-5D-3L descriptive system comprises five dimensions: mobility, self-care, usual activities, pain/discomfort, and anxiety/depression. Each dimension has 3 levels: no problems, some problems, and extreme problems with scores between 1 and 3 for each dimension. The EQ-VAS displays the patient's self-rated health on a vertical visual analogue scale with scores between 0 (Worst imaginable health state) and 100 (Best imaginable health state). The scores from the dimensions are used to calculate an index value that reflects how good or bad a health state is in relation to the general population of a country, and this facilitates the calculation of quality-adjusted life years (QALYs) that are used to inform economic evaluations of healthcare. The UK value set was used for the calculation of the EQ-5D-3L index [12].

Costs

The healthcare perspective was used for the cost analyses. Cost data were retrieved using care data registries in the three regions in Sweden where the study was performed, i.e., Östergötland, Jönköping and Kalmar. The retrieved data comprised all costs for outpatient clinic/primary care contacts and hospital admissions. Cost data were not available for six participants, who were residents in other regions, although they had been treated in one of the four recruitment hospitals and thereby included in the RCT. These patients are excluded from the analysis in this study.

Cost-effectiveness

Cost-effectiveness was assessed with an incremental cost-effectiveness ratio (ICER). The costs and effects included in the ICER are from baseline to one year post intervention, but also costs for guidance and support to patients in iCBT and ODF groups. The ICER can be

For peer review only - http://bmjopen.bmj.com/site/about/guidelines.xhtml

seen as the additional costs needed to gain an additional QALY by the iCBT compared to ODF.

Patient and public involvement

It was not possible to involve patients or the public in the design, conduct, and reporting of this study as this was mainly based on registry data.

Data analysis

Demographic data and healthcare costs were presented using numbers, percentages, mean values, and standard deviations. Chi-Square test was used for categorical variables and Student's *t*-test for continuous variables when analysing differences in demographic data between the iCBT and ODF groups.

To calculate the QALYs, missing data regarding HRQoL index value was imputed using last observation carried forward. The average index value was then multiplied by the time between each measurement (i.e., baseline to 9 weeks, 9 weeks to 6 months and 6 months to 12 months) and the one-year QALY was the sum of the four QALY values. Student´s *t*-test was then used to compare the QALY between the groups. When comparing the difference in EQ-VAS over 12 months between the groups, analysis of covariance (ANCOVA) was used which allows adjusting for baseline scores and regression to the mean.

Costs were in Swedish Kronor and were converted into Euros using the rate of May 2021 (i.e., 1 Euro = 10.50 Swedish Kronor). Costs for the iCBT and ODF were estimated based on the mean salary of a nurse (i.e., €5440) including cost surcharges, resulting in a cost per hour of €34. The time needed to guide and support patients were in total 140 hours for the iCBT group and 11 hours for the ODF group, leading to total costs of €4760 and €374, respectively. The costs for outpatient clinic/primary care contacts and hospital admissions are reported as costs for materials (including drugs and materials and medical devices), staff (including staff

For peer review only - http://bmjopen.bmj.com/site/about/guidelines.xhtml

and premises), medical services (including radiology and laboratory services), and surgery (including anesthesia, surgery, postoperative care, and intensive care). In 205 (2.3%) of a total of 8893 outpatient clinic/primary care contacts, the costs were not specified. These costs are therefore reported under staff costs since most of the costs for the outpatient/primary care contacts were related to staff costs. Cost data concern the years 2017-2019 regardless of inflation.

Cost-effectiveness as assessed by the ICER was calculated by dividing the incremental costs by the incremental effects between the groups. The ICER was bootstrapped five thousand times to plot the cost-effectiveness plane and cost-effectiveness acceptability curve to reveal the uncertainty around the results.

The IBM SPSS version 25.0 was used for data analysis. The level of $p<.05$ was used to determine statistically significant differences.

Results

Study participants

The sample in the original RCT consisted of 144 participants. Data on healthcare cost were available for 138 participants (n=70 in the iCBT group and n=68 in the ODF group), 86 men and 52 women with a mean age of 63 years (SD 12.0) (Table 1). There were no significant differences between the iCBT and ODF groups in demographic data or baseline levels of depression and HRQoL. The six participants with no healthcare cost data had significantly better baseline HRQoL (p=.023) compared to the others, but no other statistically significant differences were found in the other variables in Table 1.

For peer review only - http://bmjopen.bmj.com/site/about/guidelines.xhtml

Table 1: Demographic data of patients randomized to Internet-delivered cognitive behavioural therapy (iCBT) or online discussion forum (ODF) or excluded due to no cost data.

	iCBT (n=70)	ODF (n=68)	Excluded (n=6)
**Sex, n (%)**			
Male	46 (66)	40 (59)	3 (50)
Female	24 (34)	28 (41)	3 (50)
**Age, mean (SD)**	62 (12)	64 (12)	60 (19)
**Educational level, n (%)**			
Elementary	7 (10)	9 (13)	3 (50)
Upper secondary/high school	15 (21)	21 (31)	1 (17)
Post-secondary vocational education	12 (17)	6 (9)	0 (0)
College/university	36 (51)	32 (47)	2 (33)
**Work status, n (%)**			
Working	25 (36)	17 (25)	2 (33)
Sick leave/disability pension	7 (10)	10 (15)	1 (17)
Retired	32 (46)	33 (49)	3 (50)
Other (including unemployed and students)	6(9)	8 (12)	0 (0)
**Marital status, n (%)**			
Married/cohabiting	52 (74)	50 (74)	4 (67)
Living alone	18 (26)	18 (26)	2 (33)
**Financial Situation, n (%)**			
Very good	10 (14)	10 (15)	1 (17)
Good	48 (69)	43 (63)	3 (50)
Problematic	9 (13)	13 (19)	2 (33)
Very problematic	3 (4)	2 (3)	0 (0)
**Smoking, n (%)**			
Never	32 (46)	34 (50)	3 (50)
Ex-smoker	36 (51)	31 (46)	3 (50)
Smoker	2 (3)	3 (4)	0 (0)
**Alcohol, n (%)**			
0-4 units per week	49 (70)	54 (79)	6 (100)
5-9 units per week	17 (24)	10 (15)	0 (0)
10-14 units per week	3 (4)	4 (6)	0 (0)
15 or more units per week	1 (1)	0 (0)	0 (0)
**Depressive symptoms and HRQoL**			
PHQ-9, mean (SD)	10.6 (4.5)	10.1 (5.0)	12.9 (5.0)
EQ-VAS mean (SD)	52.8 (20.0)	56.2 (18.0)	**72.5 (13.0)***
**Type of CVD**			
Ischemic heart disease, n (%)	34 (49)	28 (41)	1 (17)
Atrial fibrillation, n (%)	38 (54)	39 (57)	4 (67)
Heart failure, n (%)	18 (26)	18 (26)	2 (33)
**Self-reported primary heart-related health problem**			
Ischemic heart disease, n (%)	26 (37)	23 (34)	0
Atrial fibrillation, n (%)	32 (46)	29 (43)	4 (67)
Heart failure, n (%)	12 (17)	16 (24)	2 (33)
**Co-morbidities (3 or more), n (%)**	10 (14)	13 (19)	0 (0)

* Significantly higher HRQoL at baseline in those excluded due to no healthcare cost data

For peer review only

Health-related quality of life and quality of adjusted years

Data on HRQoL was collected from 69 patients in the iCBT group and 67 patients in the ODF group at baseline, and from 58 and 50 patients in each group at 12-month follow-up. The iCBT group reported an improvement in EQ-VAS from 53.9 at baseline to 70.2 at 12-month follow-up, while the ODF group improved from 54.5 to 60.0 (Figure 1) during the same period. The iCBT group continued to improve, although slightly, over time, while the ODF group reported their best health status at 6-month follow-up and decreased at 12 months. The difference in EQ-VAS at 12-month follow-up was 10.2 points in favour of the iCBT group, which was statistically significant (p=.006).

The QALY values differed significantly (p=.007) between the iCBT and the ODF groups who had 0.713 and 0.598 QALYs, respectively. The mean difference of 0.115 corresponds with 42 days in best imaginable health status in favour of the iCBT group over the course of one year.

Costs

The costs for outpatient clinic/primary care contacts and hospital admissions for the iCBT group were lower than the costs for the ODF group the year before and during the intervention, but not the year after intervention, see Table 2. For the year post intervention, the cost was higher for the iCBT group regarding in-patient healthcare use. This is due to costs related to one participant who received intensive care for an extended period. However, none of the differences were statistically significant.

Figure 2 displays the total costs divided into material, staff, medical services, and surgery costs. No statistically significant differences were found between the groups in any of the areas.

For peer review only - http://bmjopen.bmj.com/site/about/guidelines.xhtml

Table 2: Healthcare costs in Euro per patient randomized to Internet-delivered cognitive behavioural therapy (iCBT) or online discussion forum (ODF) one year before, during (9 weeks) and one year post intervention.

	**In-patient costs**		**Out-patient/primary care costs**		**Total costs**		**P-value**
	**iCBT (n=70)**	**ODF (n=68)**	**iCBT (n=70)**	**ODF (n=68)**	**iCBT (n=70)**	**ODF (n=68)**	
**One year before intervention m (SD)**	3964 (8193)	5255 (9159)	3261 (2911)	3946 (3467)	7225 (9639)	9201 (10639)	.255
**9 weeks during intervention m (SD)**	445 (1431)	491 (1874)	504 (657)	620 (779)	949 (1755)	1111 (2242)	.636
**One year post intervention m (SD)**	4472 (22030)	1886 (5281)	3054 (3111)	3365 (3348)	7526 (22728)	5250 (6792)	.430

Cost-effectiveness

The cost-effectiveness results from a healthcare perspective are presented in Table 3. The ICER for iCBT versus ODF was €18,865 per QALY saved.

Table 3: Cost-effectiveness of Internet-delivered cognitive behavioural therapy (iCBT) compared to online discussion forum (ODF)

	**iCBT**	**ODF**	**Incrementell difference**
**Cost for healthcare use (Euro)**	8541.3	6366.7	2174.6
**Effect (QALY)**	0.71277	0.59750	0.11527
**Cost-effectiveness (ICER)**			18,865.3

The incremental cost-effectiveness plane and the acceptability curve for cost per QALY are presented in Figure 3. In about 24.5% of the cases the cost-effectiveness plane indicated a cheaper but more effective intervention, and in 75% a costlier and more effective intervention than ODF. Only in less than 0.5% of the cases, there was an indication of a costlier, but less effective intervention compared to ODF. The cost-effectiveness acceptability curve indicates that iCBT is cost-effective compared to ODF, with a 50% probability of the iCBT being cost-effective at a willingness to pay threshold of €15,905 per QALY.

For peer review only - http://bmjopen.bmj.com/site/about/guidelines.xhtml

Discussion

This study evaluated the cost-effectiveness of iCBT compared to ODF in patients with CVD and depressive symptoms. We found that iCBT led to sustained improvement in HRQoL at 12-month follow-up. Although there are no definite criterions to define what is to be considered as cost-effective in the Swedish healthcare system, the ICER of €18,865 can be seen as cost-effective in comparison with the cost-effectiveness threshold range of (£20,000-£30,000, or converted to Euro approximately €23,400-€35,100) as proposed by the NICE guidelines [13].

The cost-effectiveness of iCBT as a treatment for depression has been reported in a systematic review of 12 studies [14]. Seven of the reviewed studies reported an ICER for the intervention ranging between €3,088 and €22,609 and were deemed as cost-effective compared to the control condition according to the NICE threshold range [13]. At the 12-month follow-up we found significantly better HRQoL and consequently better QALYs in the iCBT group. Our ICER of €18,865 can be compared with other iCBT studies [14] and thus be seen as cost-effective. Moreover, we also found a 50% probability that iCBT compared to ODF was cost-effective at a willingness to pay threshold of €15,905 per QALY. This result is similar to another iCBT study treating depression in patients with diabetes mellitus and reporting a 51% probability of cost-effectiveness to a willingness to pay of €14,000 [15].

At the 12-month follow-up we could however not detect any significant reduction in healthcare costs of iCBT compared to ODF. Patients with CVD and depressive symptoms seem to be more reluctant to perform self-care and to seek care when needed [16]. On the other hand, improvement in depressive symptoms in patients with CVD has been found to be associated with an increase in self-care [17]. Thus, one explanation is that patients became less depressed following iCBT and by this more active in their own self-care, for example

For peer review only - http://bmjopen.bmj.com/site/about/guidelines.xhtml

seeking healthcare when that was needed. Another explanation for not finding any differences between the groups in healthcare costs could be because patients in the ODF group also were offered iCBT post intervention due to ethical reasons, and 38 % of them accepted and took part of the intervention. This may have impacted the possibility to detect differences between the groups at the 12-month follow-up.

In the review by Paganini et al [14], the mean age of the populations in 11 of the 12 studies ranged between 30-50 years and none of the populations were included based on having any somatic disease. Our studied CVD patients with a mean age of 63 years are at higher risk than younger patients without a somatic disease to suffer a deterioration in their physical health despite improvement in depressive symptoms by iCBT or not. After scrutinizing the costs, we found one participant in the iCBT group who had received intensive care for an extended period to a cost of € 200,000. A psychological intervention (e.g., iCBT) may not be expected to have any major effect on the pathophysiologic progress of the CVD compared to pharmacological (e.g., angiotensin receptor neprilisyn inhibitor) or technical interventions (e.g., pacemaker). Therefore, another explanation could be that when evaluating cost-effectiveness of psychosocial interventions in patients with chronic somatic diseases one may have to expect a limited possibility to detect reductions in healthcare costs.

Cardiac rehabilitation (CR) can be seen as the cornerstone in the management of CVD with the goal to improve quality of life and to reduce morbidity and mortality in these patients [18]. This is achieved by supervision and education to teach patients to improve their self-care and to modify CVD risk factors by, for example, stop smoking, eating healthy diets, and becoming physically active. Depression is another modifiable risk factor in CVD [19]. Despite this, a recent systematic review of 19 studies studying the cost-effectiveness of CR reported that psychological interventions were the least studied CR components and they concluded that today there is limited evidence for the cost-effectiveness of such interventions

For peer review only - http://bmjopen.bmj.com/site/about/guidelines.xhtml

in CR [6]. Moreover, to our knowledge no studies have evaluated the cost-effectiveness of Internet-based psychological interventions aimed to treat depressive symptoms in patients with CVD. It is therefore not easy to compare our data with other studies in CVD. However, four studies included in the review of Shields et al [6] evaluated the cost-effectiveness of CR as a telehealth intervention. They reported ICERs for CR as telehealth between dominant and €21,200 for three of the studies, which were deemed to be cost-effective. The fourth study had an ICER of €512,198 and was therefore not cost-effective. Our ICER of €18,865 for iCBT to treat depressive symptoms in patients with CVD is comparable to the cost-effectiveness for CR performed as telehealth.

Guided iCBT seems to be more effective than unguided iCBT [14]. Furthermore, an additional advantage when treating patients with CVD is if iCBT is guided by healthcare personnel with experience in CVD care, such as nurses, and a brief education in CBT [1]. This could certainly facilitate the implementation of iCBT in CVD care. A drawback with guidance is that policymakers at first sight can see this to drive the costs for delivering iCBT. On the other hand, Sheilds et al [6] discussed that it is more likely that the costs will be reduced when scaling up a telehealth intervention in a clinical setting, for example that initial investments can be spread over a larger number of patients. To summarize, we have in previous studies reported that iCBT improves depressive symptoms as well as physical activity and HRQoL in patients with CVD [1, 20]. This study also shows that iCBT is a cost-effective risk factor modification tool that can be used in the clinical management of depressive symptoms in patients with CVD.

Limitations

A major limitation with this study is that it was primarily designed to evaluate the effect of iCBT on depression, and thus not designed for health economic evaluation. However, it is not

For peer review only - http://bmjopen.bmj.com/site/about/guidelines.xhtml

uncommon that health-economic evaluations are performed as secondary data analysis of RCT studies. It is therefore possible that the health-economic benefits of iCBT have been underestimated in our study.

Conclusions

iCBT for depression in CVD patients can be cost-effective and implementation into CR could be feasible. However, before realization more studies evaluating treatment and cost-effectiveness of iCBT in CVD patients are needed.

Contributors

GM, JL, GA and PJ contributed to the conception and design of the study. GM, JL and MH collected and processed the data, and performed statistical analyses. GM, JL, GA, MH and PJ contributed to the analysis and interpretation of the data and drafting of the manuscript.

Funding

The study was funded by the Swedish Research Council (2015-02600) and the Medical Research of Southeast Sweden (FORSS-848511).

Competing interest

There are no competing interests to declare.

Ethical approval and consent to participate

The study was approved by the Regional Ethical Review Board in Linköping, Sweden (ref. no. 2011/166-31). The primary study was registered at ClinicalTrials.gov (NCT02778074). Participants signed an informed consent before study inclusion.

For peer review only - http://bmjopen.bmj.com/site/about/guidelines.xhtml

For peer review only

Data availability statement

The datasets used and/or analyzed are available from the corresponding author on reasonable request.

For peer review only

For peer review only - http://bmjopen.bmj.com/site/about/guidelines.xhtml

References

1. Johansson P, Westas M, Andersson G, Alehagen U, Broström A, Jaarsma T, Mourad G, Lundgren J: **An Internet-Based Cognitive Behavioral Therapy Program Adapted to Patients With Cardiovascular Disease and Depression: Randomized Controlled Trial**. *JMIR Ment Health* 2019, **6**(10):e14648.
2. Hare DL, Toukhsati SR, Johansson P, Jaarsma T: **Depression and cardiovascular disease: a clinical review**. *Eur Heart J* 2014, **35**(21):1365-1372.
3. Davydow DS, Fenger-Grøn M, Ribe AR, Pedersen HS, Prior A, Vedsted P, Unützer J, Vestergaard M: **Depression and risk of hospitalisations and rehospitalisations for ambulatory care-sensitive conditions in Denmark: a population-based cohort study**. *BMJ Open* 2015, **5**(12):e009878.
4. Szpakowski N, Qiu F, Masih S, Kurdyak P, Wijeysundera HC: **Economic Impact of Subsequent Depression in Patients With a New Diagnosis of Stable Angina: A Population-Based Study**. *J Am Heart Assoc* 2017, **6**(10).
5. Palacios J, Khondoker M, Mann A, Tylee A, Hotopf M: **Depression and anxiety symptom trajectories in coronary heart disease: Associations with measures of disability and impact on 3-year health care costs**. *J Psychosom Res* 2018, **104**:1-8.
6. Shields GE, Wells A, Doherty P, Heagerty A, Buck D, Davies LM: **Cost-effectiveness of cardiac rehabilitation: a systematic review**. *Heart* 2018, **104**(17):1403-1410.
7. Johansson P, Andersson G, Jaarsma T, Lundgren J, Westas M, Mourad G: **Psychological distress in patients with cardiovascular disease: time to do something about it?** *Eur J Cardiovasc Nurs* 2021, **20**(4):293-294.
8. Reavell J, Hopkinson M, Clarkesmith D, Lane DA: **Effectiveness of Cognitive Behavioral Therapy for Depression and Anxiety in Patients With Cardiovascular Disease: A Systematic Review and Meta-Analysis**. *Psychosom Med* 2018, **80**(8):742-753.
9. Andrews G, Basu A, Cuijpers P, Craske MG, McEvoy P, English CL, Newby JM: **Computer therapy for the anxiety and depression disorders is effective, acceptable and practical health care: An updated meta-analysis**. *J Anxiety Disord* 2018, **55**:70-78.
10. Lundgren JG, Dahlström Ö, Andersson G, Jaarsma T, Kärner Köhler A, Johansson P: **The Effect of Guided Web-Based Cognitive Behavioral Therapy on Patients With Depressive Symptoms and Heart Failure: A Pilot Randomized Controlled Trial**. *J Med Internet Res* 2016, **18**(8):e194.
11. Schneider LH, Hadjistavropoulos HD, Dear BF, Titov N: **Efficacy of internet-delivered cognitive behavioural therapy following an acute coronary event: A randomized controlled trial**. *Internet Interv* 2020, **21**:100324.
12. EuroQolGroup. **EQ-5D**. 2017-11-06; Available from: [www.euroqolgroup.org]
13. McCabe C, Claxton K, Culyer AJ: **The NICE cost-effectiveness threshold: what it is and what that means**. *Pharmacoeconomics* 2008, **26**(9):733-744.
14. Paganini S, Teigelkötter W, Buntrock C, Baumeister H: **Economic evaluations of internet- and mobile-based interventions for the treatment and prevention of depression: A systematic review**. *J Affect Disord* 2018, **225**:733-755.
15. Nobis S, Ebert DD, Lehr D, Smit F, Buntrock C, Berking M, Baumeister H, Snoek F, Funk B, Riper H: **Web-based intervention for depressive symptoms in adults with types 1 and 2 diabetes mellitus: a health economic evaluation**. *Br J Psychiatry* 2018, **212**(4):199-206.

For peer review only - http://bmjopen.bmj.com/site/about/guidelines.xhtml

16. Johansson P, Nieuwenhuis M, Lesman-Leegte I, van Veldhuisen DJ, Jaarsma T: **Depression and the delay between symptom onset and hospitalization in heart failure patients**. *Eur J Heart Fail* 2011, **13**(2):214-219.
17. Johansson P, Jaarsma T, Andersson G, Lundgren J: **The impact of internet-based cognitive behavioral therapy and depressive symptoms on self-care behavior in patients with heart failure. A secondary analysis of a randomised controlled trial**. *Int J Nurs Stud* 2019:103454.
18. Visseren FLJ, Mach F, Smulders YM, Carballo D, Koskinas KC, Bäck M, Benetos A, Biffi A, Boavida JM, Capodanno D *et al*: **2021 ESC Guidelines on cardiovascular disease prevention in clinical practice**. *Eur Heart J* 2021, **42**(34):3227-3337.
19. Yusuf S, Hawken S, Ounpuu S, Dans T, Avezum A, Lanas F, McQueen M, Budaj A, Pais P, Varigos J *et al*: **Effect of potentially modifiable risk factors associated with myocardial infarction in 52 countries (the INTERHEART study): case-control study**. *Lancet* 2004, **364**(9438):937-952.
20. Johansson P, Svensson E, Andersson G, Lundgren J: **Trajectories and associations between depression and physical activity in patients with cardiovascular disease during participation in an internet-based cognitive behavioural therapy programme**. *European Journal of Cardiovascular Nursing* 2020, **20**(2):124-131.

For peer review only

For peer review only - http://bmjopen.bmj.com/site/about/guidelines.xhtml

Figure 1: EQ-VAS measured before and 9 weeks, 6 and 12 months after the intervention in the Internet-delivered cognitive behavioural therapy (iCBT) and online discussion forum (ODF) groups.

Figure 2: Mean cost per patient randomized to Internet-delivered cognitive behavioural therapy (iCBT, n=70) or online discussion forum (ODF, n=68) one year before, during (9 weeks) and one year post intervention.

Figure 3: Cost-effectiveness plane and acceptability curve for cost per QALY

For peer review only

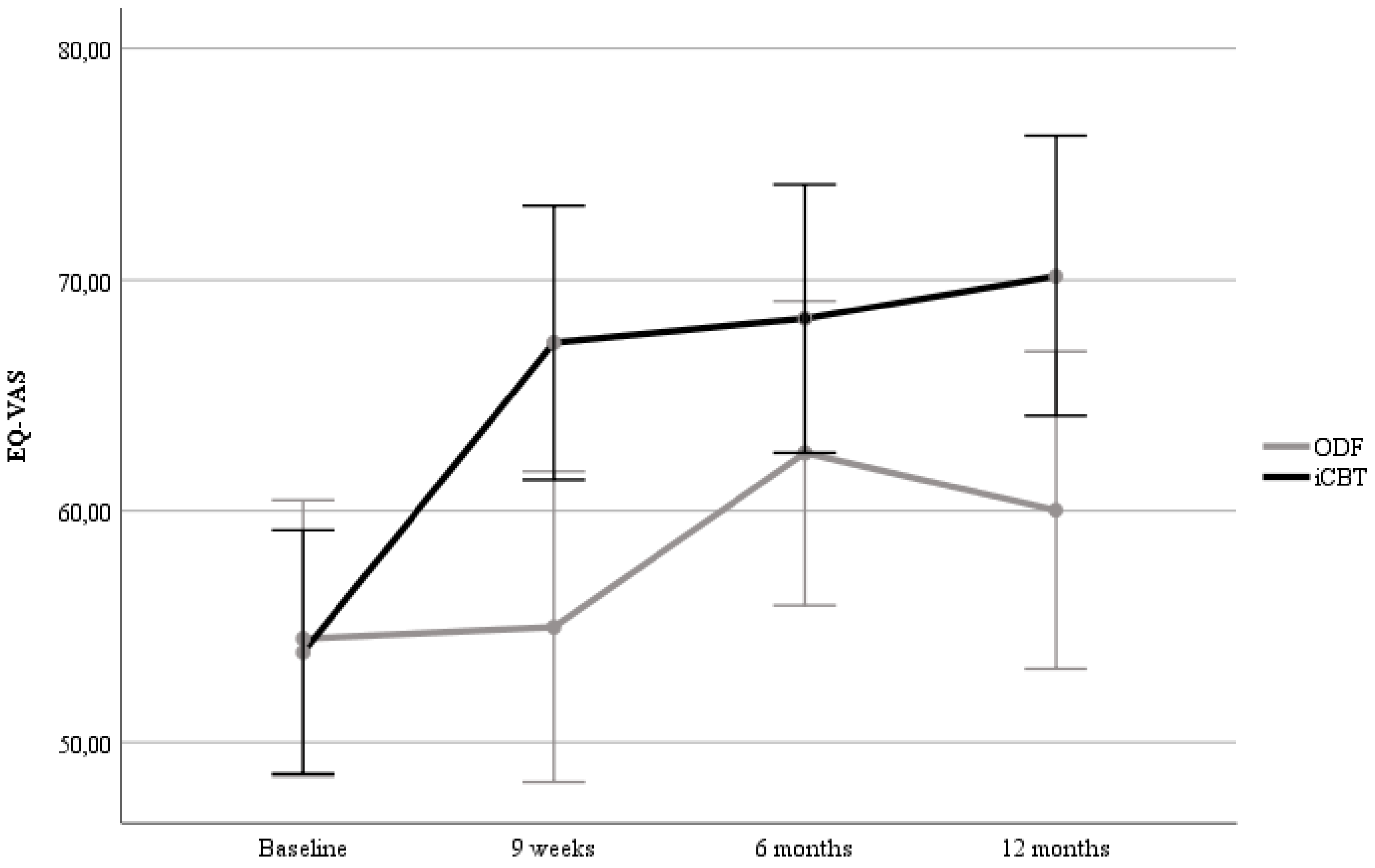
80,00
70,00
60,00
50,00
EQ-VAS
Baseline
9 weeks
6 months
12 months
ODF
iCBT

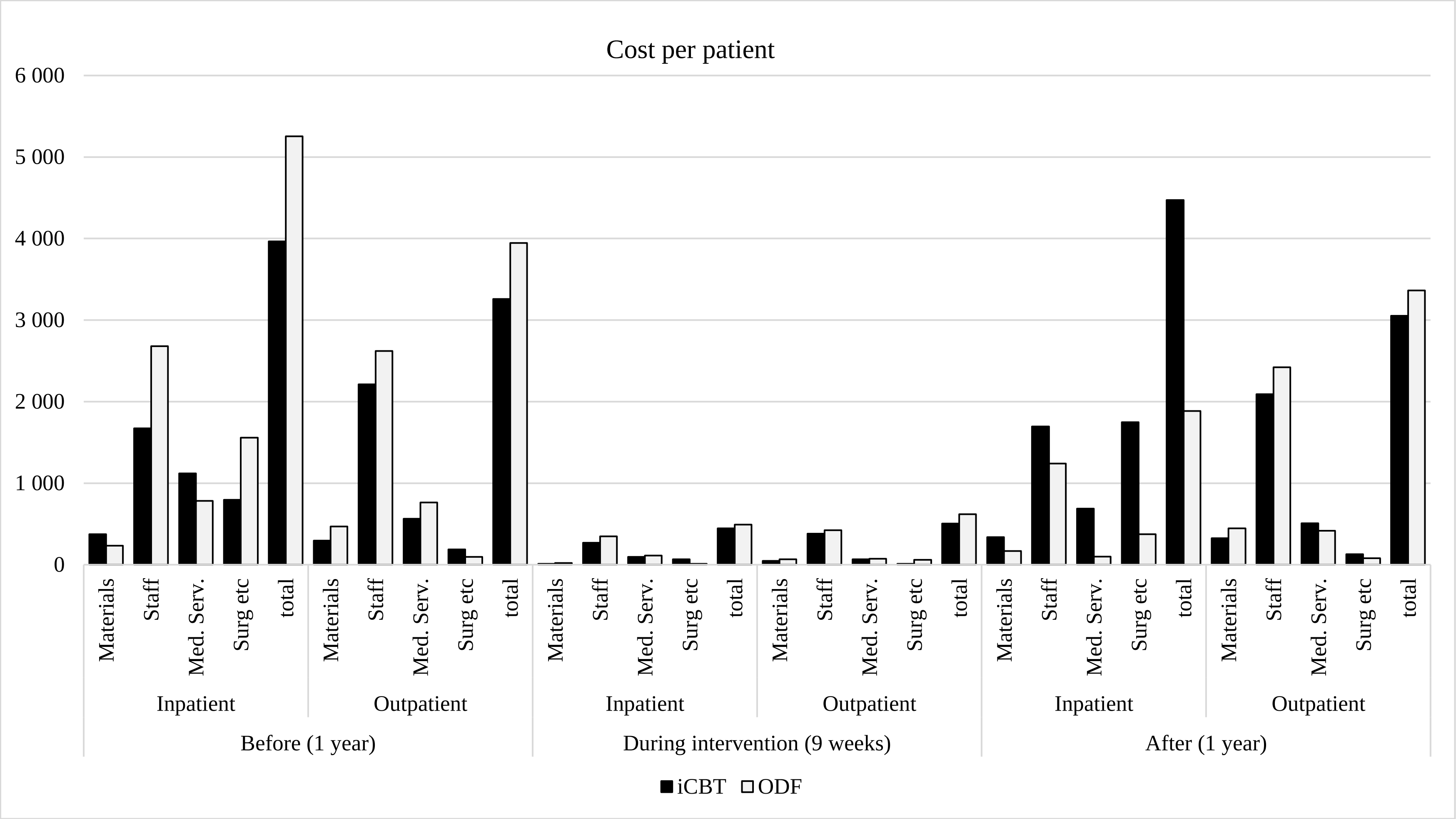
Cost per patient
6 000
5 000
4 000
3 000
2 000
1 000
0
Materials
Staff
Med. Serv.
Surg etc
total
Inpatient
Outpatient
Before (1 year)
During intervention (9 weeks)
After (1 year)
iCBT
ODF

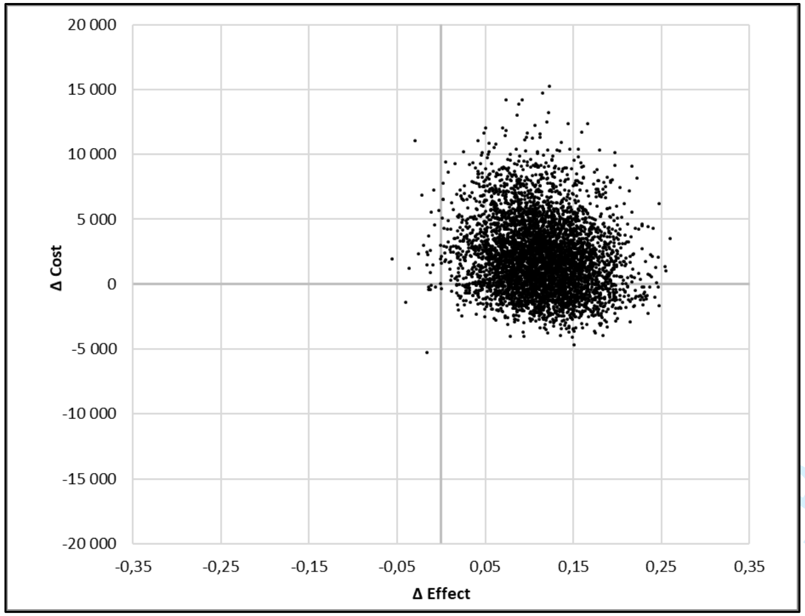
20 000
15 000
10 000
5 000
0
-5 000
-10 000
-15 000
-20 000
Δ Cost
-0,35
-0,25
-0,15
-0,05
0,05
0,15
0,25
0,35
Δ Effect

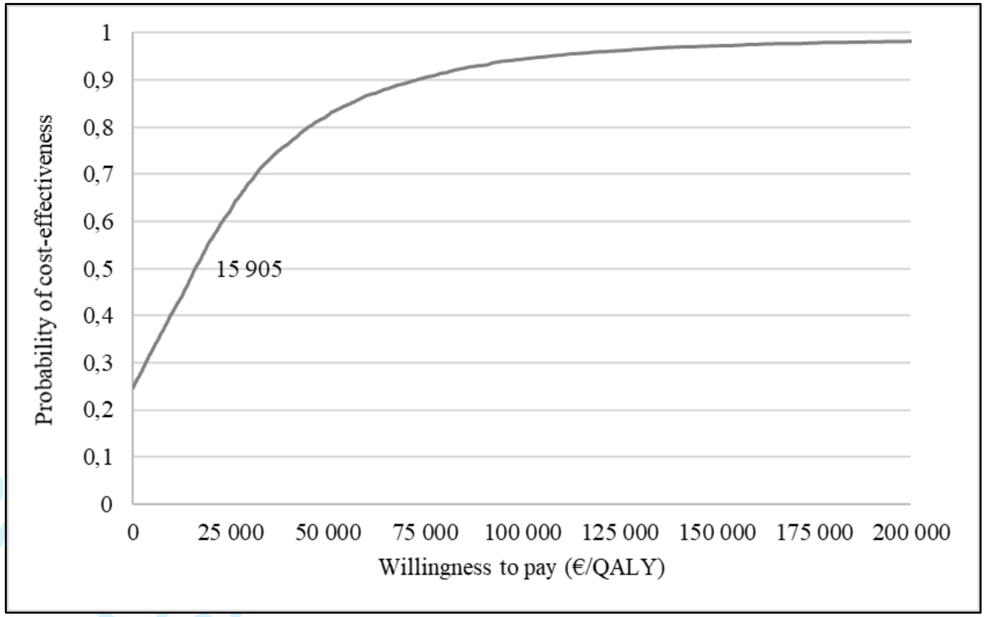
Probability of cost-effectiveness
1
0,9
0,8
0,7
0,6
0,5
0,4
0,3
0,2
0,1
0
15 905
0
25 000
50 000
75 000
100 000
125 000
150 000
175 000
200 000
Willingness to pay (€/QALY)

CONSORT 2010 checklist of information to include when reporting a randomised trial*

Section/Topic	Item No	Checklist item	Reported on page No
**Title and abstract**			
	1a	Identification as a randomised trial in the title	Title page 1
	1b	Structured summary of trial design, methods, results, and conclusions (for specific guidance see CONSORT for abstracts)	Page 2
**Introduction**			
Background and objectives	2a	Scientific background and explanation of rationale	Page 4 to 5
	2b	Specific objectives or hypotheses	Page 5
**Methods**			
Trial design	3a	Description of trial design (such as parallel, factorial) including allocation ratio	Page 5 briefly. *Explained in detail in the main reference: Johansson et al. JMIR Ment Health* 2019; 6: e14648. DOI: 10.2196/14648
	3b	Important changes to methods after trial commencement (such as eligibility criteria), with reasons	N/A
Participants	4a	Eligibility criteria for participants	Page 5 briefly. *Explained in detail in the main reference: Johansson et al. JMIR Ment Health* 2019; 6: e14648. DOI: 10.2196/14648
	4b	Settings and locations where the data were collected	Page 5 briefly.

For peer review only - http://bmjopen.bmj.com/site/about/guidelines.xhtml

			Explained in detail in the main reference: Johansson et al. JMIR Ment Health 2019; 6: e14648. DOI: 10.2196/14648
Interventions	5	The interventions for each group with sufficient details to allow replication, including how and when they were actually administered	*Page 5 briefly.* <br>*Explained in detail in the main reference: Johansson et al. JMIR Ment Health* 2019; 6: e14648. DOI: 10.2196/14648
Outcomes	6a	Completely defined pre-specified primary and secondary outcome measures, including how and when they were assessed	*Page 6-7 briefly.* <br>*Explained in detail in the main reference: Johansson et al. JMIR Ment Health* 2019; 6: e14648. DOI: 10.2196/14648
	6b	Any changes to trial outcomes after the trial commenced, with reasons	N/A
Sample size	7a	How sample size was determined	*Explained in detail in the main reference: Johansson et*

For peer review only - http://bmjopen.bmj.com/site/about/guidelines.xhtml

			al. JMIR Ment Health 2019; 6: e14648. DOI: 10.2196/14648
	7b	When applicable, explanation of any interim analyses and stopping guidelines	N/A
Randomisation:			
Sequence generation	8a	Method used to generate the random allocation sequence	*Explained in detail in the main reference: Johansson et al. JMIR Ment Health* 2019; 6: e14648. DOI: 10.2196/14648
	8b	Type of randomisation; details of any restriction (such as blocking and block size)	*Explained in detail in the main reference: Johansson et al. JMIR Ment Health* 2019; 6: e14648. DOI: 10.2196/14648
Allocation concealment mechanism	9	Mechanism used to implement the random allocation sequence (such as sequentially numbered containers), describing any steps taken to conceal the sequence until interventions were assigned	*Explained in detail in the main reference: Johansson et al. JMIR Ment Health* 2019; 6: e14648. DOI: 10.2196/14648
Implementation	10	Who generated the random allocation sequence, who enrolled participants, and who assigned participants to interventions	*Explained in detail in the main reference:*

For peer review only

For peer review only - http://bmjopen.bmj.com/site/about/guidelines.xhtml

			Johansson et al. JMIR Ment Health 2019; 6: e14648. DOI: 10.2196/14648
Blinding	11a	If done, who was blinded after assignment to interventions (for example, participants, care providers, those assessing outcomes) and how	N/A
	11b	If relevant, description of the similarity of interventions	N/A
Statistical methods	12a	Statistical methods used to compare groups for primary and secondary outcomes	Page 7-8
	12b	Methods for additional analyses, such as subgroup analyses and adjusted analyses	Page 7-8
**Results**			
Participant flow (a diagram is strongly recommended)	13a	For each group, the numbers of participants who were randomly assigned, received intended treatment, and were analysed for the primary outcome	*Explained in detail in the main reference: Johansson et al. JMIR Ment Health* 2019; 6: e14648. DOI: 10.2196/14648
	13b	For each group, losses and exclusions after randomisation, together with reasons	*Explained in detail in the main reference: Johansson et al. JMIR Ment Health* 2019; 6: e14648. DOI: 10.2196/14648
Recruitment	14a	Dates defining the periods of recruitment and follow-up	*Explained in detail in the main reference: Johansson et al. JMIR Ment Health* 2019; 6:

For peer review only - http://bmjopen.bmj.com/site/about/guidelines.xhtml

			e14648. DOI: 10.2196/14648
	14b	Why the trial ended or was stopped	N/A
Baseline data	15	A table showing baseline demographic and clinical characteristics for each group	Described in text page 8 and Table page 9. *Explained in detail in the main reference: Johansson et al. JMIR Ment Health* 2019; 6: e14648. DOI: 10.2196/14648
Numbers analysed	16	For each group, number of participants (denominator) included in each analysis and whether the analysis was by original assigned groups	Page 10 briefly. *Explained in detail in the main reference: Johansson et al. JMIR Ment Health* 2019; 6: e14648. DOI: 10.2196/14648
Outcomes and estimation	17a	For each primary and secondary outcome, results for each group, and the estimated effect size and its precision (such as 95% confidence interval)	*Page 10-12. Explained in detail in the main reference: Johansson et al. JMIR Ment Health* 2019; 6: e14648. DOI: 10.2196/14648

For peer review only

	17b	For binary outcomes, presentation of both absolute and relative effect sizes is recommended	N/A
Ancillary analyses	18	Results of any other analyses performed, including subgroup analyses and adjusted analyses, distinguishing pre-specified from exploratory	*Explained in detail in the main reference: Johansson et al. JMIR Ment Health* 2019; 6: e14648. DOI: 10.2196/14648
Harms	19	All important harms or unintended effects in each group (for specific guidance see CONSORT for harms)	*Explained in detail in the main reference: Johansson et al. JMIR Ment Health* 2019; 6: e14648. DOI: 10.2196/14648
**Discussion**			
Limitations	20	Trial limitations, addressing sources of potential bias, imprecision, and, if relevant, multiplicity of analyses	Page 16
Generalisability	21	Generalisability (external validity, applicability) of the trial findings	Page 13-15
Interpretation	22	Interpretation consistent with results, balancing benefits and harms, and considering other relevant evidence	Page 13-15
**Other information**			
Registration	23	Registration number and name of trial registry	Page 16
Protocol	24	Where the full trial protocol can be accessed, if available	Page 16
Funding	25	Sources of funding and other support (such as supply of drugs), role of funders	Page 16

*We strongly recommend reading this statement in conjunction with the CONSORT 2010 Explanation and Elaboration for important clarifications on all the items. If relevant, we also recommend reading CONSORT extensions for cluster randomised trials, non-inferiority and equivalence trials, non-pharmacological treatments, herbal interventions, and pragmatic trials. Additional extensions are forthcoming: for those and for up to date references relevant to this checklist, see www.consort-statement.org.

For peer review only

Cost-effectiveness of Internet-delivered cognitive behavioural therapy in patients with cardiovascular disease and depressive symptoms. Secondary analysis of a RCT.

Journal:	*BMJ Open*
Manuscript ID	bmjopen-2021-059939.R1
Article Type:	Original research
Date Submitted by the Author:	17-Feb-2022
Complete List of Authors:	Mourad, Ghassan; Linköping University, Department of health, medicine, and caring sciences<br>Lundgren, Johan ; Linköping University, Department of health, medicine, and caring sciences<br>Andersson, Gerhard; Linköping University, Department of Behavioural Sciences and Learning & Department of Biomedical and Clinical Sciences; Karolinska Institute, Department of Clinical Neuroscience<br>Husberg, Magnus; Linköping University, Department of health, medicine, and caring sciences<br>Johansson, Peter; Linköping University, Department of health, medicine, and caring sciences; Linköping University, Department of internal medicine
**Primary Subject Heading**:	Health economics
Secondary Subject Heading:	Cardiovascular medicine, Mental health, Nursing, Public health
Keywords:	Telemedicine < BIOTECHNOLOGY & BIOINFORMATICS, HEALTH ECONOMICS, Heart failure < CARDIOLOGY, Myocardial infarction < CARDIOLOGY

I, the Submitting Author has the right to grant and does grant on behalf of all authors of the Work (as defined in the below author licence), an exclusive licence and/or a non-exclusive licence for contributions from authors who are: i) UK Crown employees; ii) where BMJ has agreed a CC-BY licence shall apply, and/or iii) in accordance with the terms applicable for US Federal Government officers or employees acting as part of their official duties; on a worldwide, perpetual, irrevocable, royalty-free basis to BMJ Publishing Group Ltd ("BMJ") its licensees and where the relevant Journal is co-owned by BMJ to the co-owners of the Journal, to publish the Work in this journal and any other BMJ products and to exploit all rights, as set out in our licence.

The Submitting Author accepts and understands that any supply made under these terms is made by BMJ to the Submitting Author unless you are acting as an employee on behalf of your employer or a postgraduate student of an affiliated institution which is paying any applicable article publishing charge ("APC") for Open Access articles. Where the Submitting Author wishes to make the Work available on an Open Access basis (and intends to pay the relevant APC), the terms of reuse of such Open Access shall be governed by a Creative Commons licence – details of these licences and which Creative Commons licence will apply to this Work are set out in our licence referred to above.

Other than as permitted in any relevant BMJ Author's Self Archiving Policies, I confirm this Work has not been accepted for publication elsewhere, is not being considered for publication elsewhere and does not duplicate material already published. I confirm all authors consent to publication of this Work and authorise the granting of this licence.

For peer review only

Cost-effectiveness of Internet-delivered cognitive behavioural therapy in patients with cardiovascular disease and depressive symptoms. Secondary analysis of a RCT.

Ghassan Mourad PhD, RN*, Department of Health, Medicine and Caring Sciences, Linköping University, Linköping, Sweden. ghassan.mourad@liu.se

Johan Lundgren PhD, RN. Department of health, medicine, and caring sciences. Linköping University, Linköping, Sweden. johan.lundgren@liu.se

Gerhard Andersson, Professor, PhD. Department of Behavioural Sciences and Learning, Linköping University, Linköping, Sweden. Department of Biomedical and Clinical Sciences. Linköping University, Linköping, Sweden. Department of Clinical Neuroscience, Karolinska institutet, Stockholm, Sweden. gerhard.andersson@liu.se

Magnus Husberg, Department of health, medicine, and caring sciences. Linköping University, Linköping, Sweden. magnus.husberg@liu.se

Peter Johansson Professor, PhD, RN, Department of Health, Medicine and Caring Sciences and Department of internal medicine, Linköping University, Linköping, Sweden. peter.b.johansson@liu.se

***Corresponding author: Ghassan Mourad**

Address: Linköpings universitet, Kungsgatan 40, S-601 74 Norrköping

ORCID iD 0000-0001-9140-8922

Telephone: +46 11 363514

Fax: +46 11 125448

E-mail: ghassan.mourad@liu.se

Word count: 3122 (title page, abstract, references, figure legends, figures and tables excluded)

Number of tables and figures: 6

For peer review only

Abstract

Introduction: Cost-effectiveness evaluations of psychological interventions, such as Internet-delivered cognitive behavioural therapy (iCBT) programs, in patients with cardiovascular disease are rare. We recently reported moderate to large effect sizes on depressive symptoms in CVD outpatients following a 9-week iCBT program compared to an online discussion forum (ODF), in favour of iCBT [1]. In this paper we evaluate the cost-effectiveness of this intervention.

Methods: Cost-effectiveness analysis of a RCT (ClinicalTrials.gov, NCT02778074). The EQ-5D-3L was used to calculate quality-adjusted life years (QALYs). Data on healthcare costs were retrieved from healthcare registries.

Results: At 12-month follow-up, the QALY was significantly higher in iCBT compared to the ODF group (0.713 vs.0.598, p=0.007). The mean difference of 0.115 corresponds with 42 extra days in best imaginable health status in favour of the iCBT group over the course of one year. Incremental cost-effectiveness ratio (ICER) for iCBT versus ODF was €18,865 per QALY saved. The cost-effectiveness plane indicated that iCBT is a cheaper and more effective intervention in 24.5% of the cases, and in 75% a costlier and more effective intervention than ODF. Only in about 0.5% of the cases, there was an indication of a costlier, but less effective intervention compared to ODF.

Conclusions: The ICER of €18,865 was lower than the cost-effectiveness threshold range of €23,400-€35,100 as proposed by the NICE guidelines, suggesting that the iCBT treatment of depressive symptoms in patients with CVD is cost-effective.

For peer review only - http://bmjopen.bmj.com/site/about/guidelines.xhtml

Strengths and limitations

This is the first cost-effectiveness study of iCBT in patients with CVD and depression

The study was based on actual costs retrieved from data registries

The study was primarily designed to evaluate the effect of iCBT and not cost-effectiveness

What is already known about this subject?

iCBT has moderate to large effect on depressive symptoms in outpatient with CVD compared to online discussion forum

What does this study add?

iCBT treatment of depressive symptoms in outpatients with CVD is cost-effective in relation to online discussion forum

How might this impact on clinical practice?

iCBT can be used as a risk factor modification tool in cardiac rehabilitation of patients with CVD and depressive symptoms

Key words

Cardiovascular disease; Cost-effectiveness. ICER, Internet-delivered cognitive behavioural therapy, Online discussion forum.

For peer review only - http://bmjopen.bmj.com/site/about/guidelines.xhtml

Introduction

Depression is found in 20%–40% of patients with cardiovascular disease (CVD) [2], and has shown to shorten life expectancy [2], induce higher risk of non-fatal cardiac events and re-hospitalization, compared to CVD patients without depression [3]. Depression in CVD is also a societal problem in the way that it increases the healthcare costs. In a study [4] including patients with chronic stable angina, depression was a significant predictor of increased healthcare costs one year after index angiogram. The adjusted cumulative healthcare cost was 1.33, indicating a mean of 33% increase in one-year healthcare costs for those with depression. In another study, Palacios et al [5] measured depression over a three-year period in outpatients with coronary heart disease and found that patients with worsening or chronic depressive symptoms had more than twice the costs than patients without depressive symptoms. However, they also found that those with depressive symptoms who were detected and treated had lower costs than those who remained untreated. The authors emphasized that interventions targeting depression and healthcare costs in outpatients are warranted [5]. In addition to this, the cost-effectiveness of psychological interventions in cardiac rehabilitation (CR) programs has been reported to be the least studied CR component [6]. In conclusion, this highlights a need for studies that perform health-economic/cost-effectiveness evaluations of psychological interventions in CVD patients.

In recent years Internet-based cognitive behavioural therapy (iCBT) has received increased attention as a treatment option for depression in CVD patients [7, 8]. One reason for this is a low access to face-to face CBT. Another reason is the Covid-19 pandemic which has increased the need to deliver care by digital solutions, in particular for persons who are susceptible of a worser outcome in Covid-19, such as those with CVD [7]. In general, for middle-aged populations iCBT has been found to decrease depression [9], but only a minority of all iCBT studies have performed cost-effectiveness analyses. Moreover, in the existing

For peer review only - http://bmjopen.bmj.com/site/about/guidelines.xhtml

studies the evidence of the cost-effectiveness of iCBT on depression is mixed [1, 10, 11]. In CVD, only a few studies have evaluated the effect of iCBT on depression [5] and to our knowledge no study has evaluated the cost-effectiveness of iCBT in CVD patients. In a recent RCT we found that a 9-week iCBT-program had moderate to large effect on depressive symptoms in CVD outpatient compared to an online discussion forum (ODF), where new discussion topics were presented each week over a 9-week period [1]. The ODF was chosen as a comparator as it is recommended to use active controls in iCBT studies [12]. The aim of this study was to report the cost-effectiveness of iCBT compared to ODF.

Methods

Design, setting and participants

This was a cost-effectiveness study of a randomized controlled trial (RCT) aiming to reduce depressive symptoms in patients with CVD, and that has been described in detail elsewhere [1]. In brief, patients who had been in contact with the medical or cardiac clinics at four hospitals in Southeastern Sweden (N=11,992) were invited to participate by post. A total of 272 registered their interest and were screened for depressive symptoms (i.e., score ≥5 on the Patient Health Questionnaire-9 (PHQ-9)). In total 144 patients fulfilled the eligibility criteria of individuals age ≥18 years and had no hospitalizations during the past four weeks prior to inclusion. Patients were randomly allocated to nine weeks of iCBT (n=72) or ODF (n=72). Thirty-eight percent (n=27) of those in the ODF group received iCBT after study completion. The iCBT program was guided by study nurses and comprised of goal setting, psychoeducation, problem-solving, behavioural activation including homework assignments with weekly written feedback. In the iCBT group, 60% of the participants completed all seven modules, and 82% completed at least four. In the ODF, nine discussion topics were included and moderated by the study nurses. About 27% of those participants were active at least nine times during the intervention.

For peer review only - http://bmjopen.bmj.com/site/about/guidelines.xhtml

Health related quality of life and quality-adjusted life years

HRQoL was measured using the EQ-5D-3L (three levels), which consists of a descriptive system and the EQ visual analogue scale (EQ-VAS). The EQ-5D-3L descriptive system comprises five dimensions: mobility, self-care, usual activities, pain/discomfort, and anxiety/depression. Each dimension has 3 levels: no problems, some problems, and extreme problems with scores between 1 and 3 for each dimension. The EQ-VAS displays the patient's self-rated health on a vertical visual analogue scale with scores between 0 (Worst imaginable health state) and 100 (Best imaginable health state). The scores from the dimensions are used to calculate an index value that reflects how good or bad a health state is in relation to the general population of a country, and this facilitates the calculation of quality-adjusted life years (QALYs) that are used to inform economic evaluations of healthcare. The UK value set was used for the calculation of the EQ-5D-3L index [13].

Costs

The healthcare perspective was used for the cost analyses. Cost data were retrieved using care data registries in the three regions in Sweden where the study was performed, i.e., Östergötland, Jönköping and Kalmar. The retrieved data comprised all healthcare use and costs for outpatient clinic/primary care contacts and hospital admissions, and most private practices. The information is based on data from administrative registries where all data regarding healthcare use are stored. Six participants included in the primary RCT were residents in other regions that precluded costs data to be obtained. Hence, these patients are excluded from the analysis in this study.

Cost-effectiveness

Cost-effectiveness was assessed with an incremental cost-effectiveness ratio (ICER). The costs and effects included in the ICER are from baseline to one year post intervention, but

For peer review only - http://bmjopen.bmj.com/site/about/guidelines.xhtml

also costs for guidance and support to patients in iCBT and ODF groups. The ICER can be seen as the additional costs needed to gain an additional QALY by the iCBT compared to ODF.

Patient and public involvement

It was not possible to involve patients or the public in the design, conduct, and reporting of this study as this was mainly based on registry data.

Demographic data and healthcare costs were presented using numbers, percentages, mean values, and standard deviations. Chi-Square test was used for categorical variables and Student's *t*-test for continuous variables when analysing differences in demographic data between the iCBT and ODF groups.

To calculate the QALYs, missing data regarding HRQoL index value was imputed using last observation carried forward. The average index value was then multiplied by the time between each measurement (i.e., baseline to 9 weeks, 9 weeks to 6 months and 6 months to 12 months) and the one-year QALY was the sum of the four QALY values. Student´s *t*-test was then used to compare the QALY between the groups. When comparing the difference in EQ-VAS over 12 months between the groups, analysis of covariance (ANCOVA) was used which allows adjusting for baseline scores and regression to the mean.

Costs were in Swedish Kronor and were converted into Euros using the rate of May 2021 (i.e., 1 Euro = 10.50 Swedish Kronor). Costs for the iCBT and ODF were estimated based on the mean salary of a nurse (i.e., €5440) including cost surcharges, resulting in a cost per hour of €34. The time needed to guide and support patients were in total 140 hours for the iCBT group and 11 hours for the ODF group, leading to total costs of €4760 and €374, respectively. The costs for outpatient clinic/primary care contacts and hospital admissions are reported as costs for materials (including drugs and materials and medical devices), staff (including staff

For peer review only - http://bmjopen.bmj.com/site/about/guidelines.xhtml

and premises), medical services (including radiology and laboratory services), and surgery (including anesthesia, surgery, postoperative care, and intensive care). In 205 (2.3%) of a total of 8893 outpatient clinic/primary care contacts, the costs were not specified. These costs are therefore reported under staff costs since most of the costs for the outpatient/primary care contacts were related to staff costs. Cost data concern the years 2017-2019 regardless of inflation.

Cost-effectiveness as assessed by the ICER was calculated by dividing the incremental costs by the incremental effects between the groups. The ICER was bootstrapped five thousand times to plot the cost-effectiveness plane and cost-effectiveness acceptability curve to reveal the uncertainty around the results [14]. These were made in Microsoft Excel version 2201.

The IBM SPSS version 25.0 was used for data analysis. The level of $p<0.05$ was used to determine statistically significant differences.

Results

Study participants

The sample in the original RCT consisted of 144 participants. Data on healthcare cost were available for 138 participants (n=70 in the iCBT group and n=68 in the ODF group), 86 men and 52 women with a mean age of 63 years (SD 12.0) (Table 1). There were no significant differences between the iCBT and ODF groups in demographic data or baseline levels of depression and HRQoL. The six participants with no healthcare cost data had significantly better baseline HRQoL (p=0.023) compared to the others, but no other statistically significant differences were found in the other variables in Table 1.

For peer review only - http://bmjopen.bmj.com/site/about/guidelines.xhtml

Table 1: Demographic data of patients randomized to Internet-delivered cognitive behavioural therapy (iCBT) or online discussion forum (ODF) or excluded due to no cost data.

	iCBT (n=70)	ODF (n=68)	Excluded (n=6)
**Sex, n (%)**			
Male	46 (66)	40 (59)	3 (50)
Female	24 (34)	28 (41)	3 (50)
**Age, mean (SD)**	62 (12)	64 (12)	60 (19)
**Educational level, n (%)**			
Elementary	7 (10)	9 (13)	3 (50)
Upper secondary/high school	15 (21)	21 (31)	1 (17)
Post-secondary vocational education	12 (17)	6 (9)	0 (0)
College/university	36 (51)	32 (47)	2 (33)
**Work status, n (%)**			
Working	25 (36)	17 (25)	2 (33)
Sick leave/disability pension	7 (10)	10 (15)	1 (17)
Retired	32 (46)	33 (49)	3 (50)
Other (including unemployed and students)	6(9)	8 (12)	0 (0)
**Marital status, n (%)**			
Married/cohabiting	52 (74)	50 (74)	4 (67)
Living alone	18 (26)	18 (26)	2 (33)
**Financial Situation, n (%)**			
Very good	10 (14)	10 (15)	1 (17)
Good	48 (69)	43 (63)	3 (50)
Problematic	9 (13)	13 (19)	2 (33)
Very problematic	3 (4)	2 (3)	0 (0)
**Smoking, n (%)**			
Never	32 (46)	34 (50)	3 (50)
Ex-smoker	36 (51)	31 (46)	3 (50)
Smoker	2 (3)	3 (4)	0 (0)
**Alcohol, n (%)**			
0-4 units per week	49 (70)	54 (79)	6 (100)
5-9 units per week	17 (24)	10 (15)	0 (0)
10-14 units per week	3 (4)	4 (6)	0 (0)
15 or more units per week	1 (1)	0 (0)	0 (0)
**Depressive symptoms and HRQoL**			
PHQ-9, mean (SD)	10.6 (4.5)	10.1 (5.0)	12.9 (5.0)
EQ-VAS mean (SD)	52.8 (20.0)	56.2 (18.0)	**72.5 (13.0)***
**Type of CVD**			
Ischemic heart disease, n (%)	34 (49)	28 (41)	1 (17)
Atrial fibrillation, n (%)	38 (54)	39 (57)	4 (67)
Heart failure, n (%)	18 (26)	18 (26)	2 (33)
**Self-reported primary heart-related health problem**			
Ischemic heart disease, n (%)	26 (37)	23 (34)	0
Atrial fibrillation, n (%)	32 (46)	29 (43)	4 (67)
Heart failure, n (%)	12 (17)	16 (24)	2 (33)
**Co-morbidities (3 or more), n (%)**	10 (14)	13 (19)	0 (0)

* Significantly higher HRQoL at baseline in those excluded due to no healthcare cost data

For peer review only

For peer review only - http://bmjopen.bmj.com/site/about/guidelines.xhtml

Health-related quality of life and quality of adjusted years

Data on HRQoL was collected from 69 patients in the iCBT group and 67 patients in the ODF group at baseline, and from 58 and 50 patients in each group at 12-month follow-up. The iCBT group reported an improvement in EQ-VAS from 53.9 at baseline to 70.2 at 12-month follow-up, while the ODF group improved from 54.5 to 60.0 during the same period (Figure 1). The iCBT group continued to improve, although slightly, over time, while the ODF group reported their best health status at 6-month follow-up and decreased at 12 months. The difference in EQ-VAS at 12-month follow-up was 10.2 points in favour of the iCBT group, which was statistically significant (p=0.006).

The QALY values differed significantly (p=0.007) between the iCBT and the ODF groups who had 0.713 and 0.598 QALYs, respectively. The mean difference of 0.115 corresponds with 42 days in best imaginable health status in favour of the iCBT group over the course of one year.

Costs

The costs for hospital admissions and outpatient clinic/primary care contacts for the iCBT and the ODF groups are presented in Table 2. The hospital admission costs were lower in the iCBT group compared to the ODF group the year before (€3964 vs. €5255) and during the intervention (€445 vs. €491), but not the year after intervention (€4472 vs. €1886). Similar findings were found regarding the out-patient costs the year before (€3261 vs. €3946), as well as during the intervention (€504 vs. €620). However, the out-patient costs were also lower in the iCBT group the year after intervention (€3054 vs. €3365). None of the differences were statistically significant.

Figure 2 displays the total costs divided into material, staff, medical services, and surgery costs. No statistically significant differences were found between the groups in any of the areas.

For peer review only - http://bmjopen.bmj.com/site/about/guidelines.xhtml

Table 2: Healthcare costs in Euro per patient randomized to Internet-delivered cognitive behavioural therapy (iCBT) or online discussion forum (ODF) one year before, during (9 weeks) and one year post intervention.

	Hospital admission costs		Out-patient/primary care costs		Total costs		P-value
	iCBT (n=70)	ODF (n=68)	iCBT (n=70)	ODF (n=68)	iCBT (n=70)	ODF (n=68)	
**One year before intervention m (SD)**	3964 (8193)	5255 (9159)	3261 (2911)	3946 (3467)	7225 (9639)	9201 (10639)	0.255
**9 weeks during intervention m (SD)**	445 (1431)	491 (1874)	504 (657)	620 (779)	949 (1755)	1111 (2242)	0.636
**One year post intervention m (SD)**	4472 (22030)	1886 (5281)	3054 (3111)	3365 (3348)	7526 (22728)	5250 (6792)	0.430

Cost-effectiveness

The cost-effectiveness results from a healthcare perspective are presented in Table 3. The ICER for iCBT versus ODF was €18,865 per QALY saved.

Table 3: Cost-effectiveness of Internet-delivered cognitive behavioural therapy (iCBT) compared to online discussion forum (ODF)

	iCBT	ODF	Incrementell difference
**Cost for healthcare use (Euro)**	8541.3	6366.7	2174.6
**Effect (QALY)**	0.71277	0.59750	0.11527
**Cost-effectiveness (ICER)**			18,865.3

The incremental cost-effectiveness plane and the acceptability curve for cost per QALY are presented in Figure 3. In about 24.5% of the cases the cost-effectiveness plane indicated a cheaper but more effective intervention, and in 75% a costlier and more effective intervention than ODF. Only in less than 0.5% of the cases, there was an indication of a costlier, but less effective intervention compared to ODF. The cost-effectiveness acceptability curve indicates that iCBT is cost-effective compared to ODF, with a 50% probability of the iCBT being cost-effective at a willingness to pay threshold of €15,905 per QALY.

For peer review only - http://bmjopen.bmj.com/site/about/guidelines.xhtml

Discussion

This study evaluated the cost-effectiveness of iCBT compared to ODF in patients with CVD and depressive symptoms. We found that iCBT led to sustained improvement in HRQoL at 12-month follow-up. Although there are no definite criterions to define what is to be considered as cost-effective in the Swedish healthcare system, the ICER of €18,865 can be seen as cost-effective in comparison with the cost-effectiveness threshold range of (£20,000-£30,000, or converted to Euro approximately €23,400-€35,100) as proposed by the NICE guidelines [15].

The cost-effectiveness of iCBT as a treatment for depression has been reported in a systematic review of 12 studies [16]. Seven of the reviewed studies reported an ICER for the intervention ranging between €3,088 and €22,609 and were deemed as cost-effective compared to the control condition according to the NICE threshold range [15]. At the 12-month follow-up we found significantly better HRQoL and consequently better QALYs in the iCBT group. Our ICER of €18,865 can be compared with other iCBT studies [16] and thus be seen as cost-effective. Moreover, we also found a 50% probability that iCBT compared to ODF was cost-effective at a willingness to pay threshold of €15,905 per QALY. This result is similar to another iCBT study treating depression in patients with diabetes mellitus and reporting a 51% probability of cost-effectiveness to a willingness to pay of €14,000 [17].

At the 12-month follow-up we could however not detect any significant reduction in healthcare costs of iCBT compared to ODF. Patients with CVD and depressive symptoms seem to be more reluctant to perform self-care and to seek care when needed [18]. On the other hand, improvement in depressive symptoms in patients with CVD has been found to be associated with an increase in self-care [19]. Thus, one explanation is that patients became less depressed following iCBT and by this more active in their own self-care, for example

For peer review only - http://bmjopen.bmj.com/site/about/guidelines.xhtml

seeking healthcare when that was needed. Another explanation for not finding any differences between the groups in healthcare costs could be because patients in the ODF group also were offered iCBT post intervention due to ethical reasons, and 38 % of them accepted and took part of the intervention. This may have impacted the possibility to detect differences between the groups at the 12-month follow-up.

In the review by Paganini et al [16], the mean age of the populations in 11 of the 12 studies ranged between 30-50 years and none of the populations were included based on having any somatic disease. The CVD patients in the current study who had a mean age of 63 years are at higher risk than younger patients without a somatic disease to suffer a deterioration in their physical health despite improvement in depressive symptoms by iCBT or not. After scrutinizing the costs, we found one participant in the iCBT group who had received intensive care for an extended period to a cost of € 200,000. A psychological intervention (e.g., iCBT) may not be expected to have any major effect on the pathophysiologic progress of the CVD compared to pharmacological (e.g., angiotensin receptor neprilisyn inhibitor) or technical interventions (e.g., pacemaker). Therefore, another explanation could be that when evaluating cost-effectiveness of psychosocial interventions in patients with chronic somatic diseases one may have to expect a limited possibility to detect reductions in healthcare costs.

Cardiac rehabilitation (CR) can be seen as the cornerstone in the management of CVD with the goal to improve quality of life and to reduce morbidity and mortality in these patients [20]. This is achieved by supervision and education to teach patients to improve their self-care and to modify CVD risk factors by, for example, stop smoking, eating healthy diets, and becoming physically active. Depression is another modifiable risk factor in CVD [21]. Despite this, a recent systematic review of 19 studies studying the cost-effectiveness of CR reported that psychological interventions were the least studied CR components and they concluded that today there is limited evidence for the cost-effectiveness of such interventions

For peer review only - http://bmjopen.bmj.com/site/about/guidelines.xhtml

in CR [6]. Moreover, to our knowledge no studies have evaluated the cost-effectiveness of Internet-based psychological interventions aimed to treat depressive symptoms in patients with CVD. It is therefore not easy to compare our data with other studies in CVD. However, four studies included in the review of Shields et al [6] evaluated the cost-effectiveness of CR as a telehealth intervention. They reported ICERs for CR as telehealth between dominant and €21,200 for three of the studies, which were deemed to be cost-effective. The fourth study had an ICER of €512,198 and was therefore not cost-effective. An explanation for the huge ICER is that the study compared an intervention that was more expensive but had little incremental effect compared to the control. Our ICER of €18,865 for iCBT to treat depressive symptoms in patients with CVD is comparable to the cost-effectiveness for CR performed as telehealth.

Guided iCBT seems to be more effective than unguided iCBT [16]. Furthermore, an additional advantage when treating patients with CVD is if iCBT is guided by healthcare personnel with experience in CVD care, such as nurses, and a brief education in CBT [1]. This could certainly facilitate the implementation of iCBT in CVD care. A drawback with guidance is that policymakers at first sight can see this to drive the costs for delivering iCBT. On the other hand, Sheilds et al [6] discussed that it is more likely that the costs will be reduced when scaling up a telehealth intervention in a clinical setting, for example that initial investments can be spread over a larger number of patients. To summarize, we have in previous studies reported that iCBT improves depressive symptoms as well as physical activity and HRQoL in patients with CVD [1, 22]. This study also shows that iCBT is a cost-effective risk factor modification tool that can be used in the clinical management of depressive symptoms in patients with CVD.

For peer review only - http://bmjopen.bmj.com/site/about/guidelines.xhtml

Limitations

A major limitation with this study is that it was primarily designed to evaluate the effect of iCBT on depression, and thus not designed for health economic evaluation. However, it is not uncommon that health-economic evaluations are performed as secondary data analysis of RCT studies. It is therefore possible that the health-economic benefits of iCBT have been underestimated in our study.

Conclusions

iCBT for depression in CVD patients can be cost-effective and implementation into CR could be feasible. However, before realization more studies evaluating treatment and cost-effectiveness of iCBT in CVD patients are needed.

Contributors

GM, JL, GA and PJ contributed to the conception and design of the study. GM, JL and MH collected and processed the data, and performed statistical analyses. GM, JL, GA, MH and PJ contributed to the analysis and interpretation of the data and drafting of the manuscript.

Funding

The study was funded by the Swedish Research Council (2015-02600) and the Medical Research of Southeast Sweden (FORSS-848511).

Competing interest

There are no competing interests to declare.

For peer review only

Ethical approval and consent to participate

The study was approved by the Regional Ethical Review Board in Linköping, Sweden (ref. no. 2011/166-31). The primary study was registered at ClinicalTrials.gov (NCT02778074). Participants signed an informed consent before study inclusion.

Data availability statement

The datasets used and/or analyzed are available from the corresponding author on reasonable request.

For peer review only

For peer review only - http://bmjopen.bmj.com/site/about/guidelines.xhtml

References

1. Johansson P, Westas M, Andersson G, Alehagen U, Broström A, Jaarsma T, Mourad G, Lundgren J: **An Internet-Based Cognitive Behavioral Therapy Program Adapted to Patients With Cardiovascular Disease and Depression: Randomized Controlled Trial**. *JMIR Ment Health* 2019, **6**(10):e14648.
2. Hare DL, Toukhsati SR, Johansson P, Jaarsma T: **Depression and cardiovascular disease: a clinical review**. *Eur Heart J* 2014, **35**(21):1365-1372.
3. Davydow DS, Fenger-Grøn M, Ribe AR, Pedersen HS, Prior A, Vedsted P, Unützer J, Vestergaard M: **Depression and risk of hospitalisations and rehospitalisations for ambulatory care-sensitive conditions in Denmark: a population-based cohort study**. *BMJ Open* 2015, **5**(12):e009878.
4. Szpakowski N, Qiu F, Masih S, Kurdyak P, Wijeysundera HC: **Economic Impact of Subsequent Depression in Patients With a New Diagnosis of Stable Angina: A Population-Based Study**. *J Am Heart Assoc* 2017, **6**(10).
5. Palacios J, Khondoker M, Mann A, Tylee A, Hotopf M: **Depression and anxiety symptom trajectories in coronary heart disease: Associations with measures of disability and impact on 3-year health care costs**. *J Psychosom Res* 2018, **104**:1-8.
6. Shields GE, Wells A, Doherty P, Heagerty A, Buck D, Davies LM: **Cost-effectiveness of cardiac rehabilitation: a systematic review**. *Heart* 2018, **104**(17):1403-1410.
7. Johansson P, Andersson G, Jaarsma T, Lundgren J, Westas M, Mourad G: **Psychological distress in patients with cardiovascular disease: time to do something about it?** *Eur J Cardiovasc Nurs* 2021, **20**(4):293-294.
8. Reavell J, Hopkinson M, Clarkesmith D, Lane DA: **Effectiveness of Cognitive Behavioral Therapy for Depression and Anxiety in Patients With Cardiovascular Disease: A Systematic Review and Meta-Analysis**. *Psychosom Med* 2018, **80**(8):742-753.
9. Andrews G, Basu A, Cuijpers P, Craske MG, McEvoy P, English CL, Newby JM: **Computer therapy for the anxiety and depression disorders is effective, acceptable and practical health care: An updated meta-analysis**. *J Anxiety Disord* 2018, **55**:70-78.
10. Lundgren JG, Dahlström Ö, Andersson G, Jaarsma T, Kärner Köhler A, Johansson P: **The Effect of Guided Web-Based Cognitive Behavioral Therapy on Patients With Depressive Symptoms and Heart Failure: A Pilot Randomized Controlled Trial**. *J Med Internet Res* 2016, **18**(8):e194.
11. Schneider LH, Hadjistavropoulos HD, Dear BF, Titov N: **Efficacy of internet-delivered cognitive behavioural therapy following an acute coronary event: A randomized controlled trial**. *Internet Interv* 2020, **21**:100324.
12. McCombie A, Gearry R, Andrews J, Mikocka-Walus A, Mulder R: **Computerised cognitive behavioural therapy for psychological distress in patients with physical illnesses: a systematic review**. *J Clin Psychol Med Settings* 2015, **22**(1):20-44.
13. EuroQolGroup. **EQ-5D**. 2017-11-06; Available from: [www.euroqolgroup.org].
14. Drummond MF, Sculpher MJ, Claxton K, Stoddart GL, Torrance GW: **Methods for the economic evaluation of health care programmes**, Fourth edition. edn: Oxford University Press; 2015.
15. McCabe C, Claxton K, Culyer AJ: **The NICE cost-effectiveness threshold: what it is and what that means**. *Pharmacoeconomics* 2008, **26**(9):733-744.
16. Paganini S, Teigelkötter W, Buntrock C, Baumeister H: **Economic evaluations of internet- and mobile-based interventions for the treatment and prevention of depression: A systematic review**. *J Affect Disord* 2018, **225**:733-755.
17. Nobis S, Ebert DD, Lehr D, Smit F, Buntrock C, Berking M, Baumeister H, Snoek F, Funk B, Riper H: **Web-based intervention for depressive symptoms in adults with types 1 and 2 diabetes mellitus: a health economic evaluation**. *Br J Psychiatry* 2018, **212**(4):199-206.

For peer review only - http://bmjopen.bmj.com/site/about/guidelines.xhtml

18. Johansson P, Nieuwenhuis M, Lesman-Leegte I, van Veldhuisen DJ, Jaarsma T: **Depression and the delay between symptom onset and hospitalization in heart failure patients**. *Eur J Heart Fail* 2011, **13**(2):214-219.
19. Johansson P, Jaarsma T, Andersson G, Lundgren J: **The impact of internet-based cognitive behavioral therapy and depressive symptoms on self-care behavior in patients with heart failure. A secondary analysis of a randomised controlled trial**. *Int J Nurs Stud* 2019:103454.
20. Visseren FLJ, Mach F, Smulders YM, Carballo D, Koskinas KC, Bäck M, Benetos A, Biffi A, Boavida JM, Capodanno D *et al*: **2021 ESC Guidelines on cardiovascular disease prevention in clinical practice**. *Eur Heart J* 2021, **42**(34):3227-3337.
21. Yusuf S, Hawken S, Ounpuu S, Dans T, Avezum A, Lanas F, McQueen M, Budaj A, Pais P, Varigos J *et al*: **Effect of potentially modifiable risk factors associated with myocardial infarction in 52 countries (the INTERHEART study): case-control study**. *Lancet* 2004, **364**(9438):937-952.
22. Johansson P, Svensson E, Andersson G, Lundgren J: **Trajectories and associations between depression and physical activity in patients with cardiovascular disease during participation in an internet-based cognitive behavioural therapy programme**. *European Journal of Cardiovascular Nursing* 2020, **20**(2):124-131.

For peer review only

Figure 1: EQ-VAS measured before and 9 weeks, 6 and 12 months after the intervention in the Internet-delivered cognitive behavioural therapy (iCBT) and online discussion forum (ODF) groups.

Figure 2: Mean cost per patient randomized to Internet-delivered cognitive behavioural therapy (iCBT, n=70) or online discussion forum (ODF, n=68) one year before, during (9 weeks) and one year post intervention.

Figure 3: Cost-effectiveness plane and acceptability curve for cost per QALY

For peer review only

For peer review only - http://bmjopen.bmj.com/site/about/guidelines.xhtml

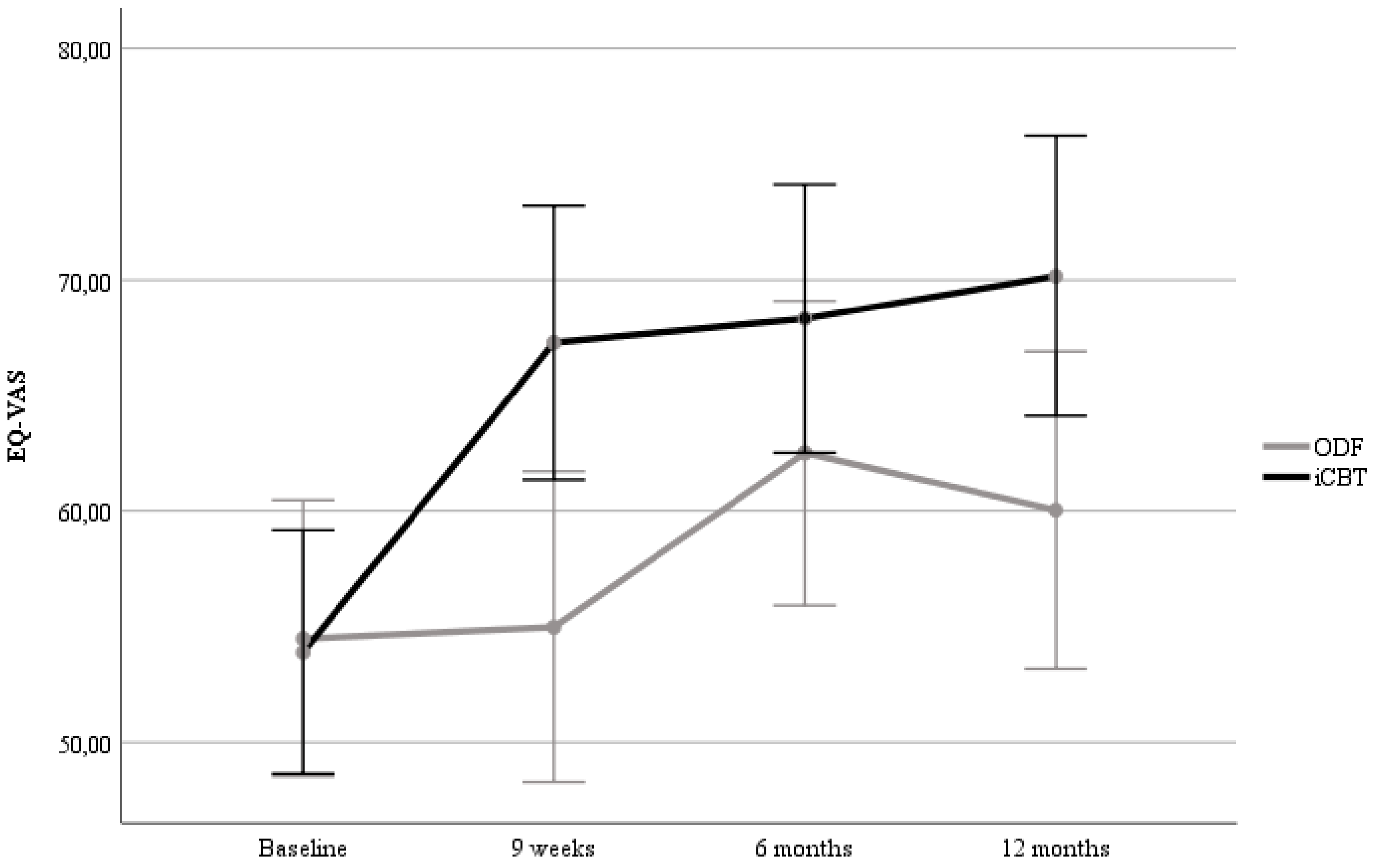
80,00
70,00
60,00
50,00
EQ-VAS
Baseline
9 weeks
6 months
12 months
ODF
iCBT

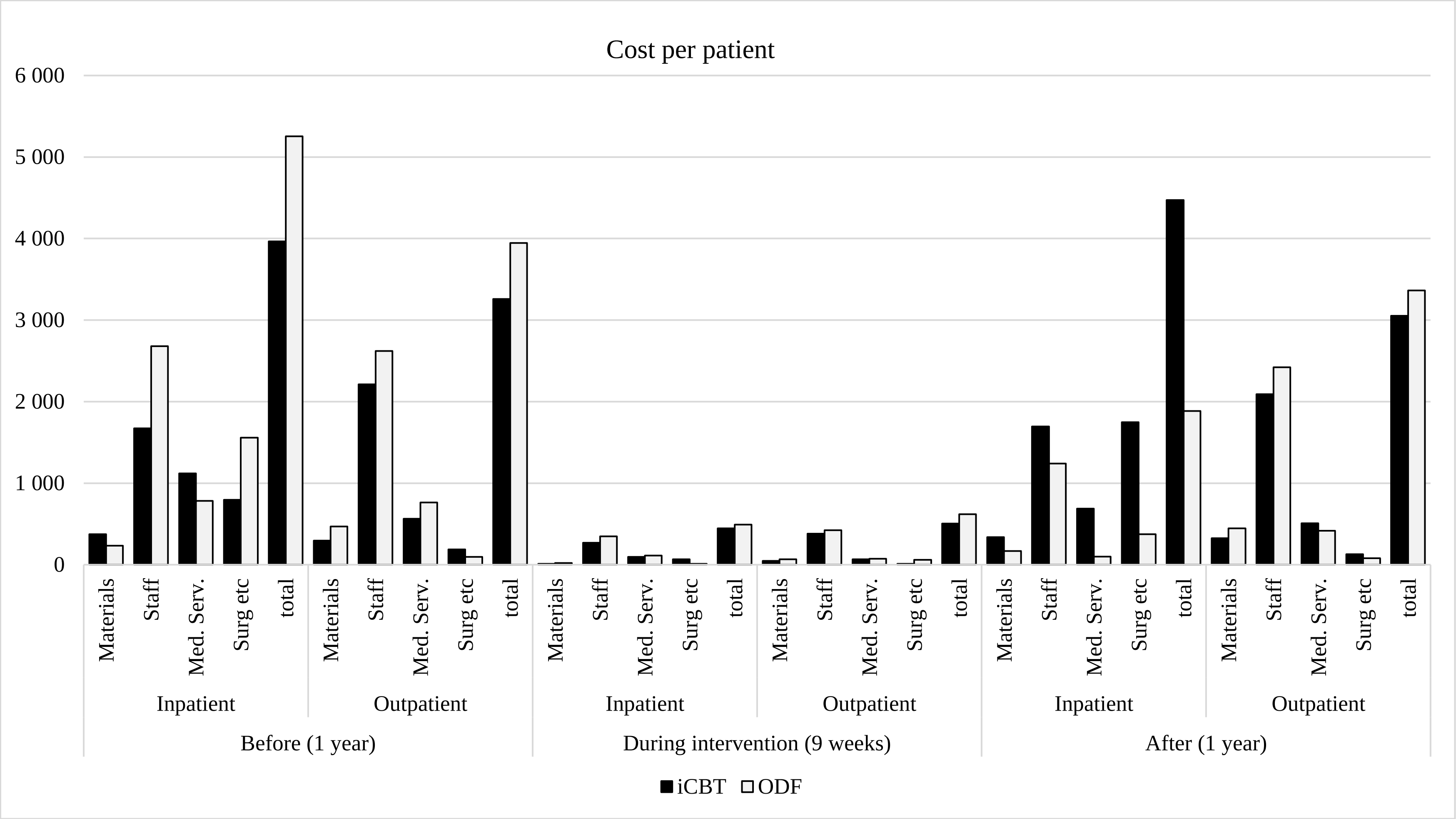
Cost per patient
6 000
5 000
4 000
3 000
2 000
1 000
0
Materials
Staff
Med. Serv.
Surg etc
total
Inpatient
Outpatient
Before (1 year)
During intervention (9 weeks)
After (1 year)
iCBT
ODF

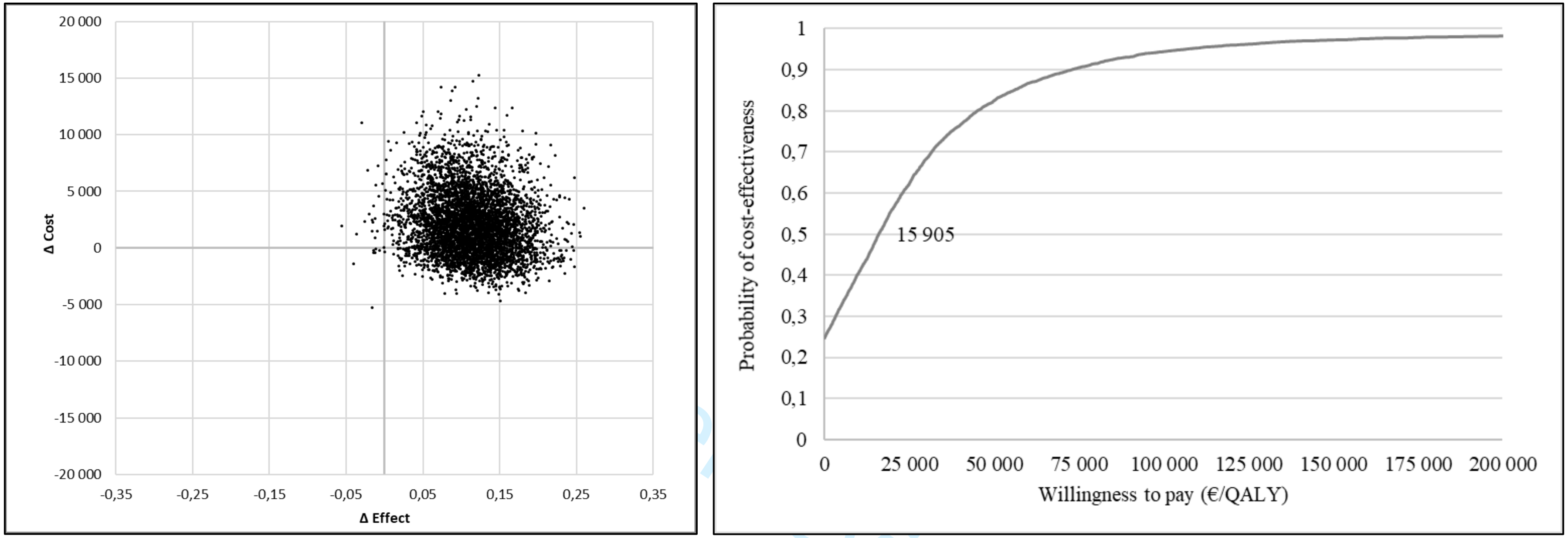
20 000
15 000
10 000
5 000
0
-5 000
-10 000
-15 000
-20 000
Δ Cost
-0,35
-0,25
-0,15
-0,05
0,05
0,15
0,25
0,35
Δ Effect
1
0,9
0,8
0,7
0,6
0,5
0,4
0,3
0,2
0,1
0
Probability of cost-effectiveness
15 905
0
25 000
50 000
75 000
100 000
125 000
150 000
175 000
200 000
Willingness to pay (€/QALY)

Consolidated Health Economic Evaluation Reporting Standards (CHEERS) 2022 Checklist

The CHEERS 2022 statement replaces the 2013 CHEERS statement, which should no longer be used. The CHEERS 2022 checklist contains 28 items with accompanying descriptions. Checklist users should indicate the section of the manuscript where relevant information can be found. The authors recommend using a section heading with a paragraph number. If an item does not apply to a particular economic evaluation, checklist users are encouraged to report "Not Applicable." If information is otherwise not reported, checklist users are encouraged to write, "Not Reported." Users should avoid the term "Not Conducted" as CHEERS is intended to guide and capture reporting. Additional information on CHEERS 2022 can be found here.

Title

1. Title

Identify the study as an economic evaluation and specify the interventions being compared.

____

Abstract

2. Abstract
Provide a structured summary that highlights context, key methods, results, and alternative analyses.

____

Introduction

3. Introduction: Background and Objectives
Give the context for the study, the study question, and its practical relevance for decision making in policy or practice.

____

Methods

4. Health economic analysis plan

© 2022 ISPOR—The Professional Society for Health Economics and Outcomes Research
International Society for Pharmacoeconomics and Outcomes Research, Inc
For peer review only - http://bmjopen.bmj.com/site/about/guidelines.xhtml

Indicate whether a health economic analysis plan was developed and where available.

5. Study population

Describe characteristics of the study population (such as age range, demographics, socioeconomic, or clinical characteristics).

6. Setting and location

Provide relevant contextual information that may influence findings.

7. Comparators

Describe the interventions or strategies being compared and why chosen.

8. Perspective

State the perspective(s) adopted by the study and why chosen.

9. Time horizon

State the time horizon for the study and why appropriate.

10. Discount rate

Report the discount rate(s) and reason chosen.

11. Selection of outcomes

Describe what outcomes were used as the measure(s) of benefit(s) and harm(s).

12. Measurement of outcomes

Describe how outcomes used to capture benefit(s) and harm(s) were measured.

© 2022 ISPOR—The Professional Society for Health Economics and Outcomes Research
International Society for Pharmacoeconomics and Outcomes Research, Inc
For peer review only - http://bmjopen.bmj.com/site/about/guidelines.xhtml

ISPOR
Improving healthcare decisions

13. Valuation of outcomes
Describe the population and methods used to measure and value outcomes.

____

14. Measurement and valuation of resources and costs
Describe how costs were valued.

____

15. Currency, price date, and conversion
Report the dates of the estimated resource quantities and unit costs, plus the currency and year of conversion.

---

16. Rationale and description of model
If modeling is used, describe in detail and why used. Report if the model is publicly available and where it can be accessed.

____

17. Analytics and assumptions
Describe any methods for analyzing or statistically transforming data, any extrapolation methods, and approaches for validating any model used.

____

18. Characterizing heterogeneity
Describe any methods used for estimating how the results of the study vary for subgroups.

____

19. Characterizing distributional effects
Describe how impacts are distributed across different individuals or adjustments made to reflect priority populations.

____

20. Characterizing uncertainty
Describe methods to characterize any sources of uncertainty in the analysis.

____ **21.**

21. Approach to engagement with patients and others affected by the study

© 2022 ISPOR—The Professional Society for Health Economics and Outcomes Research
International Society for Pharmacoeconomics and Outcomes Research, Inc
For peer review only - http://bmjopen.bmj.com/site/about/guidelines.xhtml

Describe any approaches to engage patients or service recipients, the general public, communities, or stakeholders (eg, clinicians or payers) in the design of the study.

____

Results

22. Study parameters

Report all analytic inputs (eg, values, ranges, references) including uncertainty or distributional assumptions.

____

23. Summary of main results

Report the mean values for the main categories of costs and outcomes of interest and summarize them in the most appropriate overall measure.

____

24. Effect of uncertainty

Describe how uncertainty about analytic judgments, inputs, or projections affects findings. Report the effect of choice of discount rate and time horizon, if applicable.

____

25. Effect of engagement with patients and others affected by the study

Report on any difference patient/service recipient, general public, community, or stakeholder involvement made to the approach or findings of the study.

____

Discussion

26. Study findings, limitations, generalizability, and current knowledge

Report key findings, limitations, ethical, or equity considerations not captured and how these could impact patients, policy, or practice.

____

© 2022 ISPOR—The Professional Society for Health Economics and Outcomes Research
International Society for Pharmacoeconomics and Outcomes Research, Inc
For peer review only - http://bmjopen.bmj.com/site/about/guidelines.xhtml

Other Relevant Information

27. Source of funding

Describe how the study was funded and any role of the funder in the identification, design, conduct, and reporting of the analysis.

___

28. Conflicts of interest

Report authors' conflicts of interest according to journal or International Committee of Medical Journal Editors requirements.

___

For peer review only

© 2022 ISPOR—The Professional Society for Health Economics and Outcomes Research

International Society for Pharmacoeconomics and Outcomes Research, Inc

For peer review only - http://bmjopen.bmj.com/site/about/guidelines.xhtml

CONSORT 2010 checklist of information to include when reporting a randomised trial*

Section/Topic	Item No	Checklist item	Reported on page No
**Title and abstract**			
	1a	Identification as a randomised trial in the title	Title page 1
	1b	Structured summary of trial design, methods, results, and conclusions (for specific guidance see CONSORT for abstracts)	Page 2
**Introduction**			
Background and objectives	2a	Scientific background and explanation of rationale	Page 4 to 5
	2b	Specific objectives or hypotheses	Page 5
**Methods**			
Trial design	3a	Description of trial design (such as parallel, factorial) including allocation ratio	Page 5 briefly. *Explained in detail in the main reference: Johansson et al. JMIR Ment Health* 2019; 6: e14648. DOI: 10.2196/14648
	3b	Important changes to methods after trial commencement (such as eligibility criteria), with reasons	N/A
Participants	4a	Eligibility criteria for participants	Page 5 briefly. *Explained in detail in the main reference: Johansson et al. JMIR Ment Health* 2019; 6: e14648. DOI: 10.2196/14648
	4b	Settings and locations where the data were collected	Page 5 briefly.

For peer review only

For peer review only - http://bmjopen.bmj.com/site/about/guidelines.xhtml

			Explained in detail in the main reference: Johansson et al. JMIR Ment Health 2019; 6: e14648. DOI: 10.2196/14648
Interventions	5	The interventions for each group with sufficient details to allow replication, including how and when they were actually administered	*Page 5 briefly.*<br><br>*Explained in detail in the main reference: Johansson et al. JMIR Ment Health* 2019; 6: e14648. DOI: 10.2196/14648
Outcomes	6a	Completely defined pre-specified primary and secondary outcome measures, including how and when they were assessed	*Page 6-7 briefly.*<br><br>*Explained in detail in the main reference: Johansson et al. JMIR Ment Health* 2019; 6: e14648. DOI: 10.2196/14648
	6b	Any changes to trial outcomes after the trial commenced, with reasons	N/A
Sample size	7a	How sample size was determined	*Explained in detail in the main reference: Johansson et*

For peer review only - http://bmjopen.bmj.com/site/about/guidelines.xhtml

			al. JMIR Ment Health 2019; 6: e14648. DOI: 10.2196/14648
	7b	When applicable, explanation of any interim analyses and stopping guidelines	N/A
Randomisation:			
Sequence generation	8a	Method used to generate the random allocation sequence	*Explained in detail in the main reference: Johansson et al. JMIR Ment Health* 2019; 6: e14648. DOI: 10.2196/14648
	8b	Type of randomisation; details of any restriction (such as blocking and block size)	*Explained in detail in the main reference: Johansson et al. JMIR Ment Health* 2019; 6: e14648. DOI: 10.2196/14648
Allocation concealment mechanism	9	Mechanism used to implement the random allocation sequence (such as sequentially numbered containers), describing any steps taken to conceal the sequence until interventions were assigned	*Explained in detail in the main reference: Johansson et al. JMIR Ment Health* 2019; 6: e14648. DOI: 10.2196/14648
Implementation	10	Who generated the random allocation sequence, who enrolled participants, and who assigned participants to interventions	*Explained in detail in the main reference:*

For peer review only

For peer review only - http://bmjopen.bmj.com/site/about/guidelines.xhtml

			Johansson et al. JMIR Ment Health 2019; 6: e14648. DOI: 10.2196/14648
Blinding	11a	If done, who was blinded after assignment to interventions (for example, participants, care providers, those assessing outcomes) and how	N/A
	11b	If relevant, description of the similarity of interventions	N/A
Statistical methods	12a	Statistical methods used to compare groups for primary and secondary outcomes	Page 7-8
	12b	Methods for additional analyses, such as subgroup analyses and adjusted analyses	Page 7-8
**Results**			
Participant flow (a diagram is strongly recommended)	13a	For each group, the numbers of participants who were randomly assigned, received intended treatment, and were analysed for the primary outcome	*Explained in detail in the main reference: Johansson et al. JMIR Ment Health* 2019; 6: e14648. DOI: 10.2196/14648
	13b	For each group, losses and exclusions after randomisation, together with reasons	*Explained in detail in the main reference: Johansson et al. JMIR Ment Health* 2019; 6: e14648. DOI: 10.2196/14648
Recruitment	14a	Dates defining the periods of recruitment and follow-up	*Explained in detail in the main reference: Johansson et al. JMIR Ment Health* 2019; 6:

For peer review only

			e14648. DOI: 10.2196/14648
	14b	Why the trial ended or was stopped	N/A
Baseline data	15	A table showing baseline demographic and clinical characteristics for each group	Described in text page 8 and Table page 9. *Explained in detail in the main reference: Johansson et al. JMIR Ment Health* 2019; 6: e14648. DOI: 10.2196/14648
Numbers analysed	16	For each group, number of participants (denominator) included in each analysis and whether the analysis was by original assigned groups	Page 10 briefly. *Explained in detail in the main reference: Johansson et al. JMIR Ment Health* 2019; 6: e14648. DOI: 10.2196/14648
Outcomes and estimation	17a	For each primary and secondary outcome, results for each group, and the estimated effect size and its precision (such as 95% confidence interval)	*Page 10-12. Explained in detail in the main reference: Johansson et al. JMIR Ment Health* 2019; 6: e14648. DOI: 10.2196/14648

For peer review only - http://bmjopen.bmj.com/site/about/guidelines.xhtml

	17b	For binary outcomes, presentation of both absolute and relative effect sizes is recommended	N/A
Ancillary analyses	18	Results of any other analyses performed, including subgroup analyses and adjusted analyses, distinguishing pre-specified from exploratory	*Explained in detail in the main reference: Johansson et al. JMIR Ment Health* 2019; 6: e14648. DOI: 10.2196/14648
Harms	19	All important harms or unintended effects in each group (for specific guidance see CONSORT for harms)	*Explained in detail in the main reference: Johansson et al. JMIR Ment Health* 2019; 6: e14648. DOI: 10.2196/14648
**Discussion**			
Limitations	20	Trial limitations, addressing sources of potential bias, imprecision, and, if relevant, multiplicity of analyses	Page 16
Generalisability	21	Generalisability (external validity, applicability) of the trial findings	Page 13-15
Interpretation	22	Interpretation consistent with results, balancing benefits and harms, and considering other relevant evidence	Page 13-15
**Other information**			
Registration	23	Registration number and name of trial registry	Page 16
Protocol	24	Where the full trial protocol can be accessed, if available	Page 16
Funding	25	Sources of funding and other support (such as supply of drugs), role of funders	Page 16

*We strongly recommend reading this statement in conjunction with the CONSORT 2010 Explanation and Elaboration for important clarifications on all the items. If relevant, we also recommend reading CONSORT extensions for cluster randomised trials, non-inferiority and equivalence trials, non-pharmacological treatments, herbal interventions, and pragmatic trials. Additional extensions are forthcoming: for those and for up to date references relevant to this checklist, see www.consort-statement.org.

For peer review only - http://bmjopen.bmj.com/site/about/guidelines.xhtml
For peer review only

Cost-effectiveness of Internet-delivered cognitive behavioural therapy in patients with cardiovascular disease and depressive symptoms. Secondary analysis of a RCT.

Journal:	*BMJ Open*
Manuscript ID	bmjopen-2021-059939.R2
Article Type:	Original research
Date Submitted by the Author:	03-Mar-2022
Complete List of Authors:	Mourad, Ghassan; Linköping University, Department of health, medicine, and caring sciences<br>Lundgren, Johan ; Linköping University, Department of health, medicine, and caring sciences<br>Andersson, Gerhard; Linköping University, Department of Behavioural Sciences and Learning & Department of Biomedical and Clinical Sciences; Karolinska Institute, Department of Clinical Neuroscience<br>Husberg, Magnus; Linköping University, Department of health, medicine, and caring sciences<br>Johansson, Peter; Linköping University, Department of health, medicine, and caring sciences; Linköping University, Department of internal medicine
**Primary Subject Heading**:	Health economics
Secondary Subject Heading:	Cardiovascular medicine, Mental health, Nursing, Public health
Keywords:	Telemedicine < BIOTECHNOLOGY & BIOINFORMATICS, HEALTH ECONOMICS, Heart failure < CARDIOLOGY, Myocardial infarction < CARDIOLOGY

SCHOLARONE™
Manuscripts

I, the Submitting Author has the right to grant and does grant on behalf of all authors of the Work (as defined in the below author licence), an exclusive licence and/or a non-exclusive licence for contributions from authors who are: i) UK Crown employees; ii) where BMJ has agreed a CC-BY licence shall apply, and/or iii) in accordance with the terms applicable for US Federal Government officers or employees acting as part of their official duties; on a worldwide, perpetual, irrevocable, royalty-free basis to BMJ Publishing Group Ltd ("BMJ") its licensees and where the relevant Journal is co-owned by BMJ to the co-owners of the Journal, to publish the Work in this journal and any other BMJ products and to exploit all rights, as set out in our licence.

The Submitting Author accepts and understands that any supply made under these terms is made by BMJ to the Submitting Author unless you are acting as an employee on behalf of your employer or a postgraduate student of an affiliated institution which is paying any applicable article publishing charge ("APC") for Open Access articles. Where the Submitting Author wishes to make the Work available on an Open Access basis (and intends to pay the relevant APC), the terms of reuse of such Open Access shall be governed by a Creative Commons licence – details of these licences and which Creative Commons licence will apply to this Work are set out in our licence referred to above.

Other than as permitted in any relevant BMJ Author's Self Archiving Policies, I confirm this Work has not been accepted for publication elsewhere, is not being considered for publication elsewhere and does not duplicate material already published. I confirm all authors consent to publication of this Work and authorise the granting of this licence.

For peer review only

Cost-effectiveness of Internet-delivered cognitive behavioural therapy in patients with cardiovascular disease and depressive symptoms. Secondary analysis of a RCT.

Ghassan Mourad PhD, RN*, Department of Health, Medicine and Caring Sciences, Linköping University, Linköping, Sweden. ghassan.mourad@liu.se

Johan Lundgren PhD, RN. Department of health, medicine, and caring sciences. Linköping University, Linköping, Sweden. johan.lundgren@liu.se

Gerhard Andersson, Professor, PhD. Department of Behavioural Sciences and Learning, Linköping University, Linköping, Sweden. Department of Biomedical and Clinical Sciences. Linköping University, Linköping, Sweden. Department of Clinical Neuroscience, Karolinska institutet, Stockholm, Sweden. gerhard.andersson@liu.se

Magnus Husberg, Department of health, medicine, and caring sciences. Linköping University, Linköping, Sweden. magnus.husberg@liu.se

Peter Johansson Professor, PhD, RN, Department of Health, Medicine and Caring Sciences and Department of internal medicine, Linköping University, Linköping, Sweden. peter.b.johansson@liu.se

***Corresponding author: Ghassan Mourad**

Address: Linköpings universitet, Kungsgatan 40, S-601 74 Norrköping

ORCID iD 0000-0001-9140-8922

Telephone: +46 11 363514

Fax: +46 11 125448

E-mail: ghassan.mourad@liu.se

Word count: 3122 (title page, abstract, references, figure legends, figures and tables excluded)

Number of tables and figures: 6

For peer review only

Abstract

Introduction: Cost-effectiveness evaluations of psychological interventions, such as Internet-delivered cognitive behavioural therapy (iCBT) programs, in patients with cardiovascular disease are rare. We recently reported moderate to large effect sizes on depressive symptoms in CVD outpatients following a 9-week iCBT program compared to an online discussion forum (ODF), in favour of iCBT. In this paper we evaluate the cost-effectiveness of this intervention.

Methods: Cost-effectiveness analysis of a RCT (ClinicalTrials.gov, NCT02778074). The EQ-5D-3L was used to calculate quality-adjusted life years (QALYs). Data on healthcare costs were retrieved from healthcare registries.

Results: At 12-month follow-up, the QALY was significantly higher in iCBT compared to the ODF group (0.713 vs.0.598, *p*=0.007). The mean difference of 0.115 corresponds with 42 extra days in best imaginable health status in favour of the iCBT group over the course of one year. Incremental cost-effectiveness ratio (ICER) for iCBT versus ODF was €18,865 per QALY saved. The cost-effectiveness plane indicated that iCBT is a cheaper and more effective intervention in 24.5% of the cases, and in 75% a costlier and more effective intervention than ODF. Only in about 0.5% of the cases, there was an indication of a costlier, but less effective intervention compared to ODF.

Conclusions: The ICER of €18,865 was lower than the cost-effectiveness threshold range of €23,400-€35,100 as proposed by the NICE guidelines, suggesting that the iCBT treatment of depressive symptoms in patients with CVD is cost-effective.

For peer review only

For peer review only - http://bmjopen.bmj.com/site/about/guidelines.xhtml

Strengths and limitations

This is the first cost-effectiveness study of iCBT in patients with CVD and depression

The study was based on actual costs retrieved from data registries

The study was primarily designed to evaluate the effect of iCBT and not cost-effectiveness

What is already known about this subject?

iCBT has moderate to large effect on depressive symptoms in outpatient with CVD compared to online discussion forum

What does this study add?

iCBT treatment of depressive symptoms in outpatients with CVD is cost-effective in relation to online discussion forum

How might this impact on clinical practice?

iCBT can be used as a risk factor modification tool in cardiac rehabilitation of patients with CVD and depressive symptoms

Key words

Cardiovascular disease; Cost-effectiveness. ICER, Internet-delivered cognitive behavioural therapy, Online discussion forum.

For peer review only - http://bmjopen.bmj.com/site/about/guidelines.xhtml

Introduction

Depression is found in 20%–40% of patients with cardiovascular disease (CVD) [1], and has shown to shorten life expectancy [1], induce higher risk of non-fatal cardiac events and re-hospitalization, compared to CVD patients without depression [2]. Depression in CVD is also a societal problem in the way that it increases the healthcare costs. In a study [3] including patients with chronic stable angina, depression was a significant predictor of increased healthcare costs one year after index angiogram. The adjusted cumulative healthcare cost was 1.33, indicating a mean of 33% increase in one-year healthcare costs for those with depression. In another study, Palacios et al [4] measured depression over a three-year period in outpatients with coronary heart disease and found that patients with worsening or chronic depressive symptoms had more than twice the costs than patients without depressive symptoms. However, they also found that those with depressive symptoms who were detected and treated had lower costs than those who remained untreated. The authors emphasized that interventions targeting depression and healthcare costs in outpatients are warranted [4]. In addition to this, the cost-effectiveness of psychological interventions in cardiac rehabilitation (CR) programs has been reported to be the least studied CR component [5]. In conclusion, this highlights a need for studies that perform health-economic/cost-effectiveness evaluations of psychological interventions in CVD patients.

In recent years Internet-based cognitive behavioural therapy (iCBT) has received increased attention as a treatment option for depression in CVD patients [6 7]. One reason for this is a low access to face-to face CBT. Another reason is the Covid-19 pandemic which has increased the need to deliver care by digital solutions, in particular for persons who are susceptible of a worser outcome in Covid-19, such as those with CVD [6]. In general, for middle-aged populations iCBT has been found to decrease depression [8], but only a minority of all iCBT studies have performed cost-effectiveness analyses. Moreover, in the existing studies the

For peer review only - http://bmjopen.bmj.com/site/about/guidelines.xhtml

evidence of the cost-effectiveness of iCBT on depression is mixed [9-11]. In CVD, only a few studies have evaluated the effect of iCBT on depression [4] and to our knowledge no study has evaluated the cost-effectiveness of iCBT in CVD patients. In a recent RCT we found that a 9-week iCBT-program had moderate to large effect on depressive symptoms in CVD outpatient compared to an online discussion forum (ODF), where new discussion topics were presented each week over a 9-week period [9]. The ODF was chosen as a comparator as it is recommended to use active controls in iCBT studies [12]. The aim of this study was to report the cost-effectiveness of iCBT compared to ODF.

Methods

Design, setting and participants

This was a cost-effectiveness study of a randomized controlled trial (RCT) aiming to reduce depressive symptoms in patients with CVD, and that has been described in detail elsewhere [9]. In brief, patients who had been in contact with the medical or cardiac clinics at four hospitals in Southeastern Sweden (N=11,992) were invited to participate by post. A total of 272 registered their interest and were screened for depressive symptoms (i.e., score ≥5 on the Patient Health Questionnaire-9 (PHQ-9)). In total 144 patients fulfilled the eligibility criteria of individuals age ≥18 years and had no hospitalizations during the past four weeks prior to inclusion. Patients were randomly allocated to nine weeks of iCBT (n=72) or ODF (n=72). Thirty-eight percent (n=27) of those in the ODF group received iCBT after study completion. The iCBT program was guided by study nurses and comprised of goal setting, psychoeducation, problem-solving, behavioural activation including homework assignments with weekly written feedback. In the iCBT group, 60% of the participants completed all seven modules, and 82% completed at least four. In the ODF, nine discussion topics were included and moderated by the study nurses. About 27% of those participants were active at least nine times during the intervention.

For peer review only - http://bmjopen.bmj.com/site/about/guidelines.xhtml

Health related quality of life and quality-adjusted life years

HRQoL was measured using the EQ-5D-3L (three levels), which consists of a descriptive system and the EQ visual analogue scale (EQ-VAS). The EQ-5D-3L descriptive system comprises five dimensions: mobility, self-care, usual activities, pain/discomfort, and anxiety/depression. Each dimension has 3 levels: no problems, some problems, and extreme problems with scores between 1 and 3 for each dimension. The EQ-VAS displays the patient's self-rated health on a vertical visual analogue scale with scores between 0 (Worst imaginable health state) and 100 (Best imaginable health state). The scores from the dimensions are used to calculate an index value that reflects how good or bad a health state is in relation to the general population of a country, and this facilitates the calculation of quality-adjusted life years (QALYs) that are used to inform economic evaluations of healthcare. The UK value set was used for the calculation of the EQ-5D-3L index [13].

Costs

The healthcare perspective was used for the cost analyses. Cost data were retrieved using care data registries in the three regions in Sweden where the study was performed, i.e., Östergötland, Jönköping and Kalmar. The retrieved data comprised all healthcare use and costs for outpatient clinic/primary care contacts and hospital admissions, and most private practices. The information is based on data from administrative registries where all data regarding healthcare use are stored. Six participants included in the primary RCT were residents in other regions that precluded costs data to be obtained. Hence, these patients are excluded from the analysis in this study.

Cost-effectiveness

Cost-effectiveness was assessed with an incremental cost-effectiveness ratio (ICER). The costs and effects included in the ICER are from baseline to one year post intervention, but

For peer review only - http://bmjopen.bmj.com/site/about/guidelines.xhtml

also costs for guidance and support to patients in iCBT and ODF groups. The ICER can be seen as the additional costs needed to gain an additional QALY by the iCBT compared to ODF.

Patient and public involvement

It was not possible to involve patients or the public in the design, conduct, and reporting of this study as this was mainly based on registry data.

Demographic data and healthcare costs were presented using numbers, percentages, mean values, and standard deviations. Chi-Square test was used for categorical variables and Student's *t*-test for continuous variables when analysing differences in demographic data between the iCBT and ODF groups.

To calculate the QALYs, missing data regarding HRQoL index value was imputed using last observation carried forward. The average index value was then multiplied by the time between each measurement (i.e., baseline to 9 weeks, 9 weeks to 6 months and 6 months to 12 months) and the one-year QALY was the sum of the four QALY values. Student´s *t*-test was then used to compare the QALY between the groups. When comparing the difference in EQ-VAS over 12 months between the groups, analysis of covariance (ANCOVA) was used which allows adjusting for baseline scores and regression to the mean.

Costs were in Swedish Kronor and were converted into Euros using the rate of May 2021 (i.e., 1 Euro = 10.50 Swedish Kronor). Costs for the iCBT and ODF were estimated based on the mean salary of a nurse (i.e., €5440) including cost surcharges, resulting in a cost per hour of €34. The time needed to guide and support patients were in total 140 hours for the iCBT group and 11 hours for the ODF group, leading to total costs of €4760 and €374, respectively. The costs for outpatient clinic/primary care contacts and hospital admissions are reported as costs for materials (including drugs and materials and medical devices), staff (including staff

For peer review only - http://bmjopen.bmj.com/site/about/guidelines.xhtml

and premises), medical services (including radiology and laboratory services), and surgery (including anesthesia, surgery, postoperative care, and intensive care). In 205 (2.3%) of a total of 8893 outpatient clinic/primary care contacts, the costs were not specified. These costs are therefore reported under staff costs since most of the costs for the outpatient/primary care contacts were related to staff costs. Cost data concern the years 2017-2019 regardless of inflation.

Cost-effectiveness as assessed by the ICER was calculated by dividing the incremental costs by the incremental effects between the groups. The ICER was bootstrapped five thousand times to plot the cost-effectiveness plane and cost-effectiveness acceptability curve to reveal the uncertainty around the results [14]. These were made in Microsoft Excel version 2201.

The IBM SPSS version 25.0 was used for data analysis. The level of $p<0.05$ was used to determine statistically significant differences.

Results

Study participants

The sample in the original RCT consisted of 144 participants. Data on healthcare cost were available for 138 participants (n=70 in the iCBT group and n=68 in the ODF group), 86 men and 52 women with a mean age of 63 years (SD 12.0) (Table 1). There were no significant differences between the iCBT and ODF groups in demographic data or baseline levels of depression and HRQoL. The six participants with no healthcare cost data had significantly better baseline HRQoL ($p=0.023$) compared to the others, but no other statistically significant differences were found in the other variables in Table 1.

For peer review only - http://bmjopen.bmj.com/site/about/guidelines.xhtml

Table 1: Demographic data of patients randomized to Internet-delivered cognitive behavioural therapy (iCBT) or online discussion forum (ODF) or excluded due to no cost data.

	iCBT (n=70)	ODF (n=68)	Excluded (n=6)
**Sex, n (%)**			
Male	46 (66)	40 (59)	3 (50)
Female	24 (34)	28 (41)	3 (50)
**Age, mean (SD)**	62 (12)	64 (12)	60 (19)
**Educational level, n (%)**			
Elementary	7 (10)	9 (13)	3 (50)
Upper secondary/high school	15 (21)	21 (31)	1 (17)
Post-secondary vocational education	12 (17)	6 (9)	0 (0)
College/university	36 (51)	32 (47)	2 (33)
**Work status, n (%)**			
Working	25 (36)	17 (25)	2 (33)
Sick leave/disability pension	7 (10)	10 (15)	1 (17)
Retired	32 (46)	33 (49)	3 (50)
Other (including unemployed and students)	6(9)	8 (12)	0 (0)
**Marital status, n (%)**			
Married/cohabiting	52 (74)	50 (74)	4 (67)
Living alone	18 (26)	18 (26)	2 (33)
**Financial Situation, n (%)**			
Very good	10 (14)	10 (15)	1 (17)
Good	48 (69)	43 (63)	3 (50)
Problematic	9 (13)	13 (19)	2 (33)
Very problematic	3 (4)	2 (3)	0 (0)
**Smoking, n (%)**			
Never	32 (46)	34 (50)	3 (50)
Ex-smoker	36 (51)	31 (46)	3 (50)
Smoker	2 (3)	3 (4)	0 (0)
**Alcohol, n (%)**			
0-4 units per week	49 (70)	54 (79)	6 (100)
5-9 units per week	17 (24)	10 (15)	0 (0)
10-14 units per week	3 (4)	4 (6)	0 (0)
15 or more units per week	1 (1)	0 (0)	0 (0)
**Depressive symptoms and HRQoL**			
PHQ-9, mean (SD)	10.6 (4.5)	10.1 (5.0)	12.9 (5.0)
EQ-VAS mean (SD)	52.8 (20.0)	56.2 (18.0)	**72.5 (13.0)***
**Type of CVD**			
Ischemic heart disease, n (%)	34 (49)	28 (41)	1 (17)
Atrial fibrillation, n (%)	38 (54)	39 (57)	4 (67)
Heart failure, n (%)	18 (26)	18 (26)	2 (33)
**Self-reported primary heart-related health problem**			
Ischemic heart disease, n (%)	26 (37)	23 (34)	0
Atrial fibrillation, n (%)	32 (46)	29 (43)	4 (67)
Heart failure, n (%)	12 (17)	16 (24)	2 (33)
**Co-morbidities (3 or more), n (%)**	10 (14)	13 (19)	0 (0)

* Significantly higher HRQoL at baseline in those excluded due to no healthcare cost data

For peer review only - http://bmjopen.bmj.com/site/about/guidelines.xhtml

Health-related quality of life and quality of adjusted years

Data on HRQoL was collected from 69 patients in the iCBT group and 67 patients in the ODF group at baseline, and from 58 and 50 patients in each group at 12-month follow-up. The iCBT group reported an improvement in EQ-VAS from 53.9 at baseline to 70.2 at 12-month follow-up, while the ODF group improved from 54.5 to 60.0 during the same period (Figure 1). The iCBT group continued to improve, although slightly, over time, while the ODF group reported their best health status at 6-month follow-up and decreased at 12 months. The difference in EQ-VAS at 12-month follow-up was 10.2 points in favour of the iCBT group, which was statistically significant (p=0.006).

The QALY values differed significantly (p=0.007) between the iCBT and the ODF groups who had 0.713 and 0.598 QALYs, respectively. The mean difference of 0.115 corresponds with 42 days in best imaginable health status in favour of the iCBT group over the course of one year.

Costs

The costs for hospital admissions and outpatient clinic/primary care contacts for the iCBT and the ODF groups are presented in Table 2. The hospital admission costs were lower in the iCBT group compared to the ODF group the year before (€3964 vs. €5255) and during the intervention (€445 vs. €491), but not the year after intervention (€4472 vs. €1886). Similar findings were found regarding the out-patient costs the year before (€3261 vs. €3946), as well as during the intervention (€504 vs. €620). However, the out-patient costs were also lower in the iCBT group the year after intervention (€3054 vs. €3365). None of the differences were statistically significant.

Figure 2 displays the total costs divided into material, staff, medical services, and surgery costs. No statistically significant differences were found between the groups in any of the areas.

For peer review only - http://bmjopen.bmj.com/site/about/guidelines.xhtml

Table 2: Healthcare costs in Euro per patient randomized to Internet-delivered cognitive behavioural therapy (iCBT) or online discussion forum (ODF) one year before, during (9 weeks) and one year post intervention.

	**Hospital admission costs**		**Out-patient/primary care costs**		**Total costs**		**P-value**
	**iCBT (n=70)**	**ODF (n=68)**	**iCBT (n=70)**	**ODF (n=68)**	**iCBT (n=70)**	**ODF (n=68)**	
**One year before intervention m (SD)**	3964 (8193)	5255 (9159)	3261 (2911)	3946 (3467)	7225 (9639)	9201 (10639)	0.255
**9 weeks during intervention m (SD)**	445 (1431)	491 (1874)	504 (657)	620 (779)	949 (1755)	1111 (2242)	0.636
**One year post intervention m (SD)**	4472 (22030)	1886 (5281)	3054 (3111)	3365 (3348)	7526 (22728)	5250 (6792)	0.430

Cost-effectiveness

The cost-effectiveness results from a healthcare perspective are presented in Table 3. The ICER for iCBT versus ODF was €18,865 per QALY saved.

Table 3: Cost-effectiveness of Internet-delivered cognitive behavioural therapy (iCBT) compared to online discussion forum (ODF)

	**iCBT**	**ODF**	**Incrementell difference**
**Cost for healthcare use (Euro)**	8541.3	6366.7	2174.6
**Effect (QALY)**	0.71277	0.59750	0.11527
**Cost-effectiveness (ICER)**			18,865.3

The incremental cost-effectiveness plane and the acceptability curve for cost per QALY are presented in Figure 3. In about 24.5% of the cases the cost-effectiveness plane indicated a cheaper but more effective intervention, and in 75% a costlier and more effective intervention than ODF. Only in less than 0.5% of the cases, there was an indication of a costlier, but less effective intervention compared to ODF. The cost-effectiveness acceptability curve indicates that iCBT is cost-effective compared to ODF, with a 50% probability of the iCBT being cost-effective at a willingness to pay threshold of €15,905 per QALY.

For peer review only - http://bmjopen.bmj.com/site/about/guidelines.xhtml

Discussion

This study evaluated the cost-effectiveness of iCBT compared to ODF in patients with CVD and depressive symptoms. We found that iCBT led to sustained improvement in HRQoL at 12-month follow-up. Although there are no definite criterions to define what is to be considered as cost-effective in the Swedish healthcare system, the ICER of €18,865 can be seen as cost-effective in comparison with the cost-effectiveness threshold range of (£20,000-£30,000, or converted to Euro approximately €23,400-€35,100) as proposed by the NICE guidelines [15].

The cost-effectiveness of iCBT as a treatment for depression has been reported in a systematic review of 12 studies [16]. Seven of the reviewed studies reported an ICER for the intervention ranging between €3,088 and €22,609 and were deemed as cost-effective compared to the control condition according to the NICE threshold range [15]. At the 12-month follow-up we found significantly better HRQoL and consequently better QALYs in the iCBT group. Our ICER of €18,865 can be compared with other iCBT studies [16] and thus be seen as cost-effective. Moreover, we also found a 50% probability that iCBT compared to ODF was cost-effective at a willingness to pay threshold of €15,905 per QALY. This result is similar to another iCBT study treating depression in patients with diabetes mellitus and reporting a 51% probability of cost-effectiveness to a willingness to pay of €14,000 [17].

At the 12-month follow-up we could however not detect any significant reduction in healthcare costs of iCBT compared to ODF. Patients with CVD and depressive symptoms seem to be more reluctant to perform self-care and to seek care when needed [18]. On the other hand, improvement in depressive symptoms in patients with CVD has been found to be associated with an increase in self-care [19]. Thus, one explanation is that patients became less depressed following iCBT and by this more active in their own self-care, for example seeking

For peer review only - http://bmjopen.bmj.com/site/about/guidelines.xhtml

healthcare when that was needed. Another explanation for not finding any differences between the groups in healthcare costs could be because patients in the ODF group also were offered iCBT post intervention due to ethical reasons, and 38 % of them accepted and took part of the intervention. This may have impacted the possibility to detect differences between the groups at the 12-month follow-up.

In the review by Paganini et al [16], the mean age of the populations in 11 of the 12 studies ranged between 30-50 years and none of the populations were included based on having any somatic disease. The CVD patients in the current study who had a mean age of 63 years are at higher risk than younger patients without a somatic disease to suffer a deterioration in their physical health despite improvement in depressive symptoms by iCBT or not. After scrutinizing the costs, we found one participant in the iCBT group who had received intensive care for an extended period to a cost of € 200,000. A psychological intervention (e.g., iCBT) may not be expected to have any major effect on the pathophysiologic progress of the CVD compared to pharmacological (e.g., angiotensin receptor neprilisyn inhibitor) or technical interventions (e.g., pacemaker). Therefore, another explanation could be that when evaluating cost-effectiveness of psychosocial interventions in patients with chronic somatic diseases one may have to expect a limited possibility to detect reductions in healthcare costs.

Cardiac rehabilitation (CR) can be seen as the cornerstone in the management of CVD with the goal to improve quality of life and to reduce morbidity and mortality in these patients [20]. This is achieved by supervision and education to teach patients to improve their self-care and to modify CVD risk factors by, for example, stop smoking, eating healthy diets, and becoming physically active. Depression is another modifiable risk factor in CVD [21]. Despite this, a recent systematic review of 19 studies studying the cost-effectiveness of CR reported that psychological interventions were the least studied CR components and they concluded that today there is limited evidence for the cost-effectiveness of such interventions in CR [5].

For peer review only - http://bmjopen.bmj.com/site/about/guidelines.xhtml

Moreover, to our knowledge no studies have evaluated the cost-effectiveness of Internet-based psychological interventions aimed to treat depressive symptoms in patients with CVD. It is therefore not easy to compare our data with other studies in CVD. However, four studies included in the review of Shields et al [5] evaluated the cost-effectiveness of CR as a telehealth intervention. They reported ICERs for CR as telehealth between dominant and €21,200 for three of the studies, which were deemed to be cost-effective. The fourth study had an ICER of €512,198 and was therefore not cost-effective. An explanation for the huge ICER is that the study compared an intervention that was more expensive but had little incremental effect compared to the control. Our ICER of €18,865 for iCBT to treat depressive symptoms in patients with CVD is comparable to the cost-effectiveness for CR performed as telehealth.

Guided iCBT seems to be more effective than unguided iCBT [16]. Furthermore, an additional advantage when treating patients with CVD is if iCBT is guided by healthcare personnel with experience in CVD care, such as nurses, and a brief education in CBT [9]. This could certainly facilitate the implementation of iCBT in CVD care. A drawback with guidance is that policymakers at first sight can see this to drive the costs for delivering iCBT. On the other hand, Sheilds et al [5] discussed that it is more likely that the costs will be reduced when scaling up a telehealth intervention in a clinical setting, for example that initial investments can be spread over a larger number of patients. To summarize, we have in previous studies reported that iCBT improves depressive symptoms as well as physical activity and HRQoL in patients with CVD [9 22]. This study also shows that iCBT is a cost-effective risk factor modification tool that can be used in the clinical management of depressive symptoms in patients with CVD.

For peer review only - http://bmjopen.bmj.com/site/about/guidelines.xhtml

Limitations

A major limitation with this study is that it was primarily designed to evaluate the effect of iCBT on depression, and thus not designed for health economic evaluation. However, it is not uncommon that health-economic evaluations are performed as secondary data analysis of RCT studies. It is therefore possible that the health-economic benefits of iCBT have been underestimated in our study.

Conclusions

iCBT for depression in CVD patients can be cost-effective and implementation into CR could be feasible. However, before realization more studies evaluating treatment and cost-effectiveness of iCBT in CVD patients are needed.

Contributors

GM, JL, GA and PJ contributed to the conception and design of the study. GM, JL and MH collected and processed the data, and performed statistical analyses. GM, JL, GA, MH and PJ contributed to the analysis and interpretation of the data and drafting of the manuscript.

Funding

The study was funded by the Swedish Research Council (2015-02600) and the Medical Research of Southeast Sweden (FORSS-848511).

Competing interest

There are no competing interests to declare.

For peer review only - http://bmjopen.bmj.com/site/about/guidelines.xhtml

Ethical approval and consent to participate

The study was approved by the Regional Ethical Review Board in Linköping, Sweden (ref. no. 2011/166-31). The primary study was registered at ClinicalTrials.gov (NCT02778074). Participants signed an informed consent before study inclusion.

Data availability statement

The datasets used and/or analyzed are available from the corresponding author on reasonable request.

For peer review only

For peer review only - http://bmjopen.bmj.com/site/about/guidelines.xhtml

References

1. Hare DL, Toukhsati SR, Johansson P, et al. Depression and cardiovascular disease: a clinical review. *Eur Heart J* 2014;35(21):1365-72. doi: 10.1093/eurheartj/eht462 [published Online First: 2013/11/28]
2. Davydow DS, Fenger-Grøn M, Ribe AR, et al. Depression and risk of hospitalisations and rehospitalisations for ambulatory care-sensitive conditions in Denmark: a population-based cohort study. *BMJ Open* 2015;5(12):e009878. doi: 10.1136/bmjopen-2015-009878 [published Online First: 2015/12/05]
3. Szpakowski N, Qiu F, Masih S, et al. Economic Impact of Subsequent Depression in Patients With a New Diagnosis of Stable Angina: A Population-Based Study. *J Am Heart Assoc* 2017;6(10) doi: 10.1161/jaha.117.006911 [published Online First: 2017/10/13]
4. Palacios J, Khondoker M, Mann A, et al. Depression and anxiety symptom trajectories in coronary heart disease: Associations with measures of disability and impact on 3-year health care costs. *J Psychosom Res* 2018;104:1-8. doi: 10.1016/j.jpsychores.2017.10.015 [published Online First: 2017/12/26]
5. Shields GE, Wells A, Doherty P, et al. Cost-effectiveness of cardiac rehabilitation: a systematic review. *Heart* 2018;104(17):1403-10. doi: 10.1136/heartjnl-2017-312809 [published Online First: 2018/04/15]
6. Johansson P, Andersson G, Jaarsma T, et al. Psychological distress in patients with cardiovascular disease: time to do something about it? *Eur J Cardiovasc Nurs* 2021;20(4):293-94. doi: 10.1093/eurjcn/zvab007 [published Online First: 2021/04/13]
7. Reavell J, Hopkinson M, Clarkesmith D, et al. Effectiveness of Cognitive Behavioral Therapy for Depression and Anxiety in Patients With Cardiovascular Disease: A Systematic Review and Meta-Analysis. *Psychosom Med* 2018;80(8):742-53. doi: 10.1097/psy.0000000000000626 [published Online First: 2018/10/04]
8. Andrews G, Basu A, Cuijpers P, et al. Computer therapy for the anxiety and depression disorders is effective, acceptable and practical health care: An updated meta-analysis. *J Anxiety Disord* 2018;55:70-78. doi: 10.1016/j.janxdis.2018.01.001 [published Online First: 2018/02/10]
9. Johansson P, Westas M, Andersson G, et al. An Internet-Based Cognitive Behavioral Therapy Program Adapted to Patients With Cardiovascular Disease and Depression: Randomized Controlled Trial. *JMIR Ment Health* 2019;6(10):e14648. doi: 10.2196/14648 [published Online First: 2019/10/05]
10. Lundgren JG, Dahlström Ö, Andersson G, et al. The Effect of Guided Web-Based Cognitive Behavioral Therapy on Patients With Depressive Symptoms and Heart Failure: A Pilot Randomized Controlled Trial. *J Med Internet Res* 2016;18(8):e194. doi: 10.2196/jmir.5556 [published Online First: 2016/08/05]
11. Schneider LH, Hadjistavropoulos HD, Dear BF, et al. Efficacy of internet-delivered cognitive behavioural therapy following an acute coronary event: A randomized controlled trial. *Internet Interv* 2020;21:100324. doi: 10.1016/j.invent.2020.100324 [published Online First: 2020/05/27]
12. McCombie A, Gearry R, Andrews J, et al. Computerised cognitive behavioural therapy for psychological distress in patients with physical illnesses: a systematic review. *J Clin Psychol Med Settings* 2015;22(1):20-44. doi: 10.1007/s10880-015-9420-0 [published Online First: 2015/02/11]
13. EuroQolGroup. EQ-5D 2017 [Available from: www.euroqolgroup.org accessed 2017-11-06.
14. Drummond MF, Sculpher MJ, Claxton K, et al. Methods for the economic evaluation of health care programmes. Fourth edition. ed: Oxford University Press 2015.
15. McCabe C, Claxton K, Culyer AJ. The NICE cost-effectiveness threshold: what it is and what that means. *Pharmacoeconomics* 2008;26(9):733-44. doi: 10.2165/00019053-200826090-00004 [published Online First: 2008/09/05]

For peer review only - http://bmjopen.bmj.com/site/about/guidelines.xhtml

16. Paganini S, Teigelkötter W, Buntrock C, et al. Economic evaluations of internet- and mobile-based interventions for the treatment and prevention of depression: A systematic review. *J Affect Disord* 2018;225:733-55. doi: 10.1016/j.jad.2017.07.018 [published Online First: 2017/09/20]
17. Nobis S, Ebert DD, Lehr D, et al. Web-based intervention for depressive symptoms in adults with types 1 and 2 diabetes mellitus: a health economic evaluation. *Br J Psychiatry* 2018;212(4):199-206. doi: 10.1192/bjp.2018.10 [published Online First: 2018/08/04]
18. Johansson P, Nieuwenhuis M, Lesman-Leegte I, et al. Depression and the delay between symptom onset and hospitalization in heart failure patients. *Eur J Heart Fail* 2011;13(2):214-9. doi: hfq200 [pii]

10.1093/eurjhf/hfq200 [published Online First: 2010/11/18]
19. Johansson P, Jaarsma T, Andersson G, et al. The impact of internet-based cognitive behavioral therapy and depressive symptoms on self-care behavior in patients with heart failure. A secondary analysis of a randomised controlled trial. *Int J Nurs Stud* 2019:103454. doi: 10.1016/j.ijnurstu.2019.103454 [published Online First: 2019/11/16]
20. Visseren FLJ, Mach F, Smulders YM, et al. 2021 ESC Guidelines on cardiovascular disease prevention in clinical practice. *Eur Heart J* 2021;42(34):3227-337. doi: 10.1093/eurheartj/ehab484 [published Online First: 2021/08/31]
21. Yusuf S, Hawken S, Ounpuu S, et al. Effect of potentially modifiable risk factors associated with myocardial infarction in 52 countries (the INTERHEART study): case-control study. *Lancet* 2004;364(9438):937-52.
22. Johansson P, Svensson E, Andersson G, et al. Trajectories and associations between depression and physical activity in patients with cardiovascular disease during participation in an internet-based cognitive behavioural therapy programme. *European Journal of Cardiovascular Nursing* 2020;20(2):124-31. doi: 10.1177/1474515120947250

For peer review only

For peer review only - http://bmjopen.bmj.com/site/about/guidelines.xhtml

Figure 1: EQ-VAS measured before and 9 weeks, 6 and 12 months after the intervention in the Internet-delivered cognitive behavioural therapy (iCBT) and online discussion forum (ODF) groups.

Figure 2: Mean cost per patient randomized to Internet-delivered cognitive behavioural therapy (iCBT, n=70) or online discussion forum (ODF, n=68) one year before, during (9 weeks) and one year post intervention.

Figure 3: Cost-effectiveness plane and acceptability curve for cost per QALY

For peer review only

For peer review only - http://bmjopen.bmj.com/site/about/guidelines.xhtml

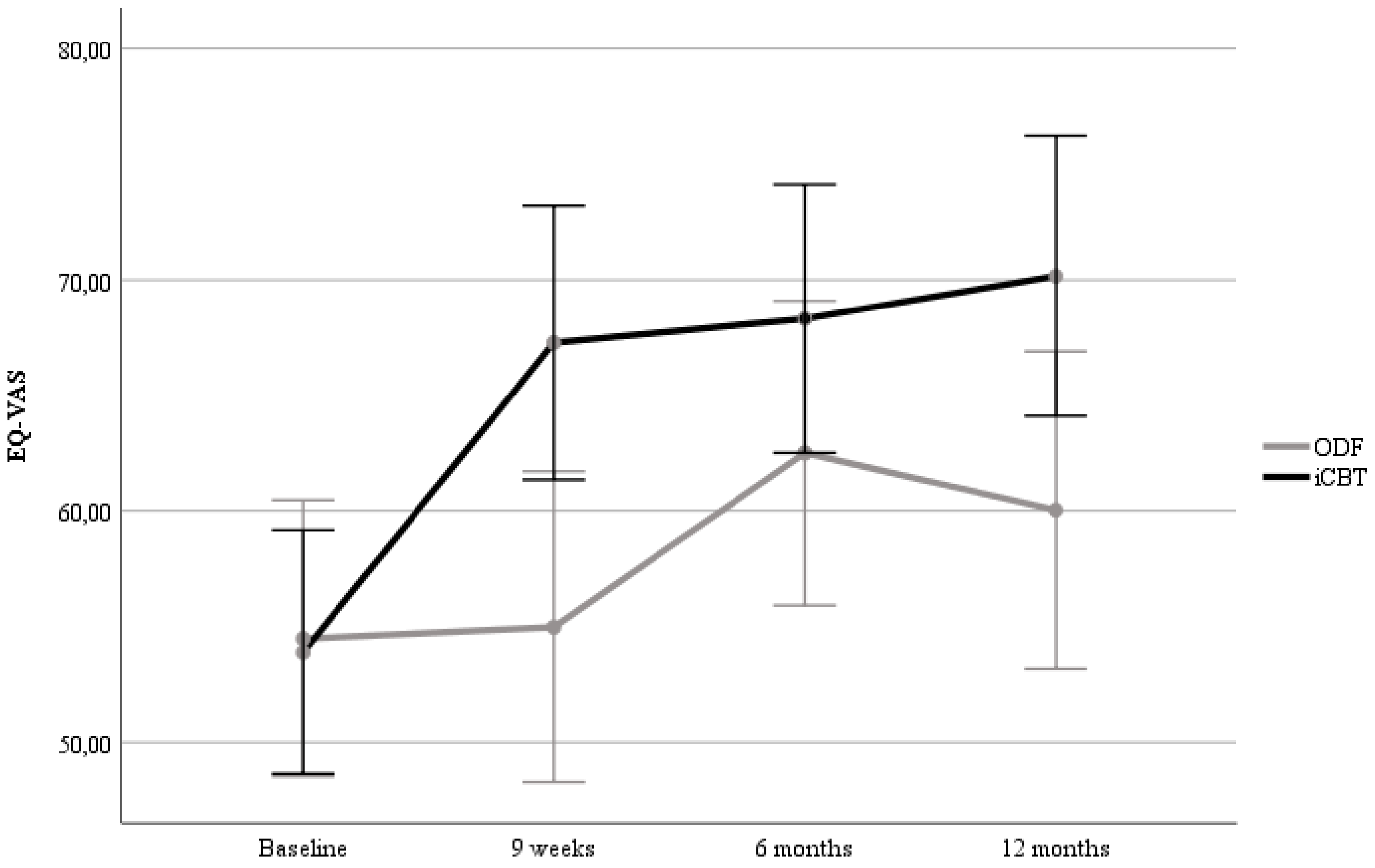

80,00
70,00
60,00
50,00
EQ-VAS
Baseline
9 weeks
6 months
12 months
ODF
iCBT

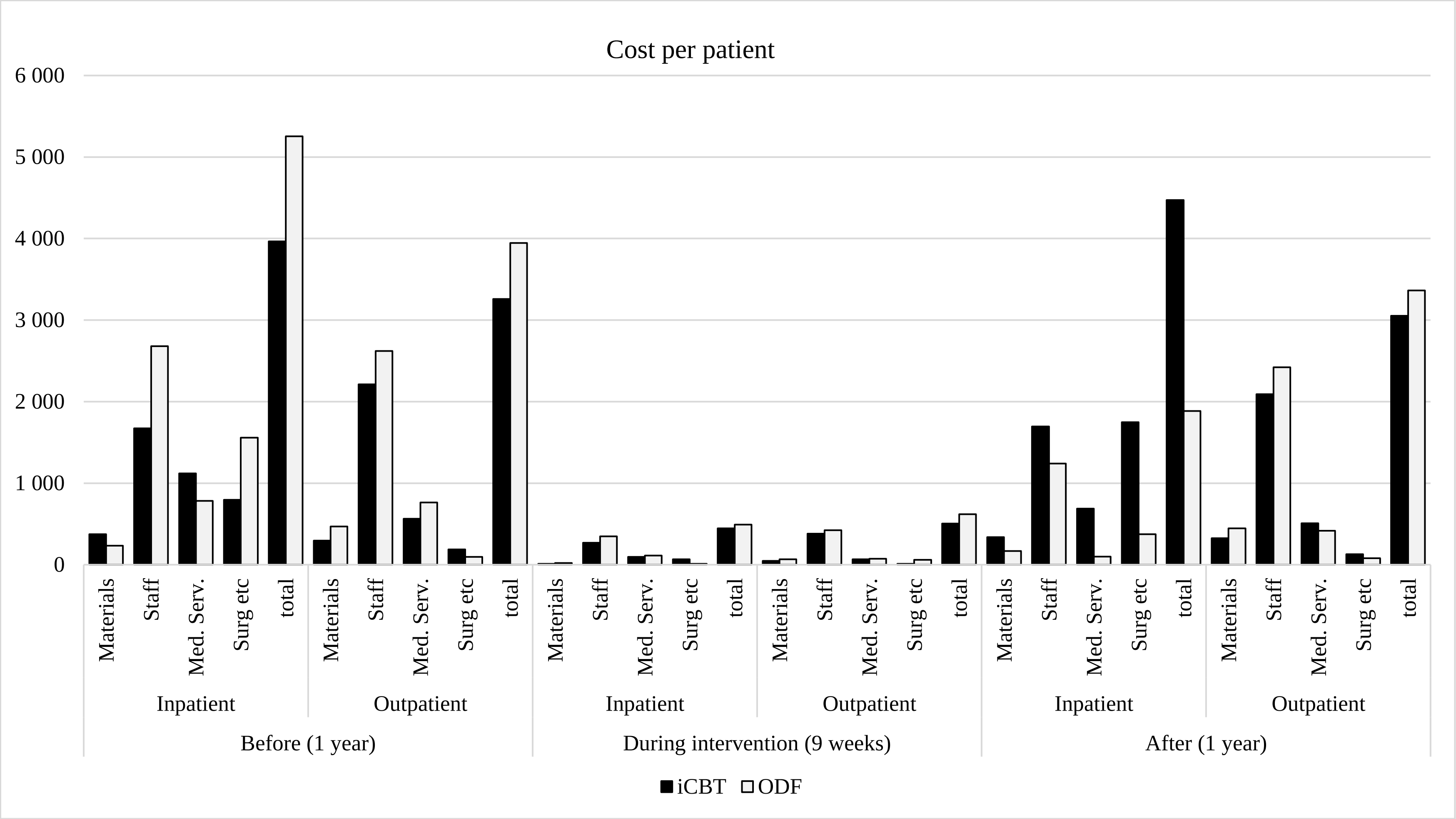
Cost per patient
6 000
5 000
4 000
3 000
2 000
1 000
0
Materials
Staff
Med. Serv.
Surg etc
total
Inpatient
Outpatient
Before (1 year)
During intervention (9 weeks)
After (1 year)
iCBT
ODF

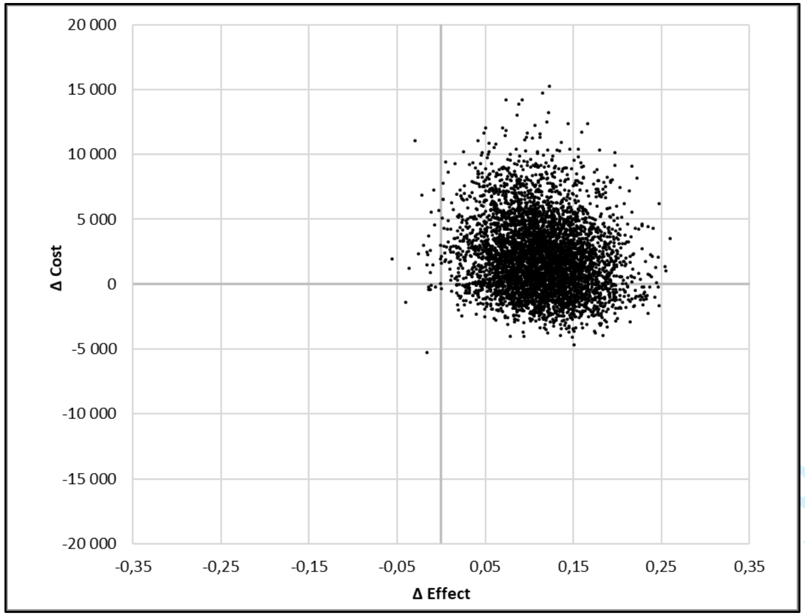
20 000
15 000
10 000
5 000
0
-5 000
-10 000
-15 000
-20 000
Δ Cost
-0,35
-0,25
-0,15
-0,05
0,05
0,15
0,25
0,35
Δ Effect

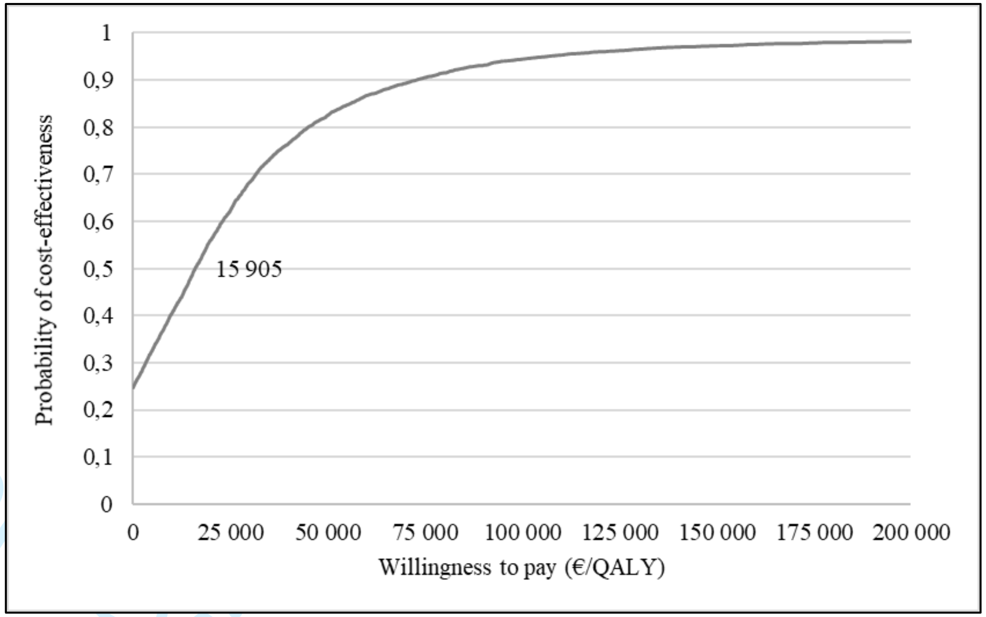
1
0,9
0,8
0,7
0,6
0,5
0,4
0,3
0,2
0,1
0
Probability of cost-effectiveness
15 905
0
25 000
50 000
75 000
100 000
125 000
150 000
175 000
200 000
Willingness to pay (€/QALY)

ISPOR
Improving healthcare decisions

Consolidated Health Economic Evaluation Reporting Standards (CHEERS) 2022 Checklist

The CHEERS 2022 statement replaces the 2013 CHEERS statement, which should no longer be used. The CHEERS 2022 checklist contains 28 items with accompanying descriptions. Checklist users should indicate the section of the manuscript where relevant information can be found. The authors recommend using a section heading with a paragraph number. If an item does not apply to a particular economic evaluation, checklist users are encouraged to report "Not Applicable." If information is otherwise not reported, checklist users are encouraged to write, "Not Reported." Users should avoid the term "Not Conducted" as CHEERS is intended to guide and capture reporting. Additional information on CHEERS 2022 can be found here.

Title

1. Title

Identify the study as an economic evaluation and specify the interventions being compared.

____

Abstract

2. Abstract

Provide a structured summary that highlights context, key methods, results, and alternative analyses.

____

Introduction

3. Introduction: Background and Objectives

Give the context for the study, the study question, and its practical relevance for decision making in policy or practice.

____

Methods

4. Health economic analysis plan

© 2022 ISPOR—The Professional Society for Health Economics and Outcomes Research
International Society for Pharmacoeconomics and Outcomes Research, Inc
For peer review only - http://bmjopen.bmj.com/site/about/guidelines.xhtml
For peer review only

Indicate whether a health economic analysis plan was developed and where available.

____

5. Study population

Describe characteristics of the study population (such as age range, demographics, socioeconomic, or clinical characteristics).

____

6. Setting and location

Provide relevant contextual information that may influence findings.

____

7. Comparators

Describe the interventions or strategies being compared and why chosen.

____

8. Perspective

State the perspective(s) adopted by the study and why chosen.

____

9. Time horizon

State the time horizon for the study and why appropriate.

____

10. Discount rate

Report the discount rate(s) and reason chosen.

---

11. Selection of outcomes

Describe what outcomes were used as the measure(s) of benefit(s) and harm(s).

____

12. Measurement of outcomes

Describe how outcomes used to capture benefit(s) and harm(s) were measured.

____

© 2022 ISPOR—The Professional Society for Health Economics and Outcomes Research

International Society for Pharmacoeconomics and Outcomes Research, Inc

For peer review only - http://bmjopen.bmj.com/site/about/guidelines.xhtml

ISPOR
Improving healthcare decisions

13. Valuation of outcomes

Describe the population and methods used to measure and value outcomes.

____

14. Measurement and valuation of resources and costs

Describe how costs were valued.

____

15. Currency, price date, and conversion

Report the dates of the estimated resource quantities and unit costs, plus the currency and year of conversion.

---

16. Rationale and description of model

If modeling is used, describe in detail and why used. Report if the model is publicly available and where it can be accessed.

____

17. Analytics and assumptions

Describe any methods for analyzing or statistically transforming data, any extrapolation methods, and approaches for validating any model used.

____

18. Characterizing heterogeneity

Describe any methods used for estimating how the results of the study vary for subgroups.

____

19. Characterizing distributional effects

Describe how impacts are distributed across different individuals or adjustments made to reflect priority populations.

____

20. Characterizing uncertainty

Describe methods to characterize any sources of uncertainty in the analysis.

____ **21.**

21. Approach to engagement with patients and others affected by the study

© 2022 ISPOR—The Professional Society for Health Economics and Outcomes Research

International Society for Pharmacoeconomics and Outcomes Research, Inc

For peer review only - http://bmjopen.bmj.com/site/about/guidelines.xhtml

Describe any approaches to engage patients or service recipients, the general public, communities, or stakeholders (eg, clinicians or payers) in the design of the study.

____

Results

22. Study parameters

Report all analytic inputs (eg, values, ranges, references) including uncertainty or distributional assumptions.

____

23. Summary of main results

Report the mean values for the main categories of costs and outcomes of interest and summarize them in the most appropriate overall measure.

____

24. Effect of uncertainty

Describe how uncertainty about analytic judgments, inputs, or projections affects findings. Report the effect of choice of discount rate and time horizon, if applicable.

____

25. Effect of engagement with patients and others affected by the study

Report on any difference patient/service recipient, general public, community, or stakeholder involvement made to the approach or findings of the study.

____

Discussion

26. Study findings, limitations, generalizability, and current knowledge

Report key findings, limitations, ethical, or equity considerations not captured and how these could impact patients, policy, or practice.

____

© 2022 ISPOR—The Professional Society for Health Economics and Outcomes Research
International Society for Pharmacoeconomics and Outcomes Research, Inc
For peer review only - http://bmjopen.bmj.com/site/about/guidelines.xhtml

Other Relevant Information

27. Source of funding

Describe how the study was funded and any role of the funder in the identification, design, conduct, and reporting of the analysis.

---

28. Conflicts of interest

Report authors' conflicts of interest according to journal or International Committee of Medical Journal Editors requirements.

For peer review only

© 2022 ISPOR—The Professional Society for Health Economics and Outcomes Research
International Society for Pharmacoeconomics and Outcomes Research, Inc
For peer review only - http://bmjopen.bmj.com/site/about/guidelines.xhtml

CONSORT 2010 checklist of information to include when reporting a randomised trial*

Section/Topic	Item No	Checklist item	Reported on page No
**Title and abstract**			
	1a	Identification as a randomised trial in the title	Title page 1
	1b	Structured summary of trial design, methods, results, and conclusions (for specific guidance see CONSORT for abstracts)	Page 2
**Introduction**			
Background and objectives	2a	Scientific background and explanation of rationale	Page 4 to 5
	2b	Specific objectives or hypotheses	Page 5
**Methods**			
Trial design	3a	Description of trial design (such as parallel, factorial) including allocation ratio	Page 5 briefly. *Explained in detail in the main reference: Johansson et al. JMIR Ment Health* 2019; 6: e14648. DOI: 10.2196/14648
	3b	Important changes to methods after trial commencement (such as eligibility criteria), with reasons	N/A
Participants	4a	Eligibility criteria for participants	Page 5 briefly. *Explained in detail in the main reference: Johansson et al. JMIR Ment Health* 2019; 6: e14648. DOI: 10.2196/14648
	4b	Settings and locations where the data were collected	Page 5 briefly.

For peer review only - http://bmjopen.bmj.com/site/about/guidelines.xhtml

			Explained in detail in the main reference: Johansson et al. JMIR Ment Health 2019; 6: e14648. DOI: 10.2196/14648
Interventions	5	The interventions for each group with sufficient details to allow replication, including how and when they were actually administered	*Page 5 briefly.*<br><br>*Explained in detail in the main reference: Johansson et al. JMIR Ment Health* 2019; 6: e14648. DOI: 10.2196/14648
Outcomes	6a	Completely defined pre-specified primary and secondary outcome measures, including how and when they were assessed	*Page 6-7 briefly.*<br><br>*Explained in detail in the main reference: Johansson et al. JMIR Ment Health* 2019; 6: e14648. DOI: 10.2196/14648
	6b	Any changes to trial outcomes after the trial commenced, with reasons	N/A
Sample size	7a	How sample size was determined	*Explained in detail in the main reference: Johansson et*

For peer review only - http://bmjopen.bmj.com/site/about/guidelines.xhtml

			al. JMIR Ment Health 2019; 6: e14648. DOI: 10.2196/14648
	7b	When applicable, explanation of any interim analyses and stopping guidelines	N/A
Randomisation:			
Sequence generation	8a	Method used to generate the random allocation sequence	*Explained in detail in the main reference: Johansson et al. JMIR Ment Health* 2019; 6: e14648. DOI: 10.2196/14648
	8b	Type of randomisation; details of any restriction (such as blocking and block size)	*Explained in detail in the main reference: Johansson et al. JMIR Ment Health* 2019; 6: e14648. DOI: 10.2196/14648
Allocation concealment mechanism	9	Mechanism used to implement the random allocation sequence (such as sequentially numbered containers), describing any steps taken to conceal the sequence until interventions were assigned	*Explained in detail in the main reference: Johansson et al. JMIR Ment Health* 2019; 6: e14648. DOI: 10.2196/14648
Implementation	10	Who generated the random allocation sequence, who enrolled participants, and who assigned participants to interventions	*Explained in detail in the main reference:*

For peer review only - http://bmjopen.bmj.com/site/about/guidelines.xhtml

			Johansson et al. JMIR Ment Health 2019; 6: e14648. DOI: 10.2196/14648
Blinding	11a	If done, who was blinded after assignment to interventions (for example, participants, care providers, those assessing outcomes) and how	N/A
	11b	If relevant, description of the similarity of interventions	N/A
Statistical methods	12a	Statistical methods used to compare groups for primary and secondary outcomes	Page 7-8
	12b	Methods for additional analyses, such as subgroup analyses and adjusted analyses	Page 7-8
**Results**			
Participant flow (a diagram is strongly recommended)	13a	For each group, the numbers of participants who were randomly assigned, received intended treatment, and were analysed for the primary outcome	*Explained in detail in the main reference: Johansson et al. JMIR Ment Health* 2019; 6: e14648. DOI: 10.2196/14648
	13b	For each group, losses and exclusions after randomisation, together with reasons	*Explained in detail in the main reference: Johansson et al. JMIR Ment Health* 2019; 6: e14648. DOI: 10.2196/14648
Recruitment	14a	Dates defining the periods of recruitment and follow-up	*Explained in detail in the main reference: Johansson et al. JMIR Ment Health* 2019; 6:

For peer review only - http://bmjopen.bmj.com/site/about/guidelines.xhtml

			e14648. DOI: 10.2196/14648
	14b	Why the trial ended or was stopped	N/A
Baseline data	15	A table showing baseline demographic and clinical characteristics for each group	Described in text page 8 and Table page 9.<br><br>*Explained in detail in the main reference: Johansson et al. JMIR Ment Health* 2019; 6: e14648. DOI: 10.2196/14648
Numbers analysed	16	For each group, number of participants (denominator) included in each analysis and whether the analysis was by original assigned groups	Page 10 briefly. *Explained in detail in the main reference: Johansson et al. JMIR Ment Health* 2019; 6: e14648. DOI: 10.2196/14648
Outcomes and estimation	17a	For each primary and secondary outcome, results for each group, and the estimated effect size and its precision (such as 95% confidence interval)	*Page 10-12. Explained in detail in the main reference: Johansson et al. JMIR Ment Health* 2019; 6: e14648. DOI: 10.2196/14648

For peer review only - http://bmjopen.bmj.com/site/about/guidelines.xhtml

	17b	For binary outcomes, presentation of both absolute and relative effect sizes is recommended	N/A
Ancillary analyses	18	Results of any other analyses performed, including subgroup analyses and adjusted analyses, distinguishing pre-specified from exploratory	*Explained in detail in the main reference: Johansson et al. JMIR Ment Health* 2019; 6: e14648. DOI: 10.2196/14648
Harms	19	All important harms or unintended effects in each group (for specific guidance see CONSORT for harms)	*Explained in detail in the main reference: Johansson et al. JMIR Ment Health* 2019; 6: e14648. DOI: 10.2196/14648
**Discussion**			
Limitations	20	Trial limitations, addressing sources of potential bias, imprecision, and, if relevant, multiplicity of analyses	Page 16
Generalisability	21	Generalisability (external validity, applicability) of the trial findings	Page 13-15
Interpretation	22	Interpretation consistent with results, balancing benefits and harms, and considering other relevant evidence	Page 13-15
**Other information**			
Registration	23	Registration number and name of trial registry	Page 16
Protocol	24	Where the full trial protocol can be accessed, if available	Page 16
Funding	25	Sources of funding and other support (such as supply of drugs), role of funders	Page 16

*We strongly recommend reading this statement in conjunction with the CONSORT 2010 Explanation and Elaboration for important clarifications on all the items. If relevant, we also recommend reading CONSORT extensions for cluster randomised trials, non-inferiority and equivalence trials, non-pharmacological treatments, herbal interventions, and pragmatic trials. Additional extensions are forthcoming: for those and for up to date references relevant to this checklist, see www.consort-statement.org.

For peer review only - http://bmjopen.bmj.com/site/about/guidelines.xhtml
